# Inflammatory imbalance and activation deficits in T cells of myasthenia gravis patients revealed by proteomic profiling

**DOI:** 10.3389/fimmu.2025.1648020

**Published:** 2025-08-04

**Authors:** Amol K. Bhandage, Anna Rostedt Punga

**Affiliations:** Clinical Neurophysiology, Department of Medical Sciences, Uppsala University, Uppsala, Sweden

**Keywords:** myasthenia gravis, CD4+, CD8+, T cells, activation, secretion, inflammatory proteins

## Abstract

Myasthenia gravis (MG) is a heterogeneous autoimmune disorder characterized by neuromuscular transmission failure and skeletal muscle fatigability, with a pathophysiology involving both cellular and humoral immune components. Despite growing interest in the immunological etiology of MG, few functional studies have addressed the role of T cells, and most existing work has focused on quantifying immune cell subsets using flow cytometry. In this study, a comparative *in vitro* analysis of resting and activated CD4+ and CD8+ T cells from MG patients and healthy controls (HC) was performed using the multiplex Proximity Extension Assay (PEA) proteomics to assess the secretion of inflammatory proteins, including cytokines and chemokines, and to define the inflammatory status of T cells in MG. Data analysis was performed using the Boruta algorithm to detect both linear and non-linear patterns, followed by multiple testing corrections, and correlation analyses. The results revealed distinct alterations in the secretion profiles of several inflammatory proteins in MG compared to HC across both T cell subsets, regardless of activation state. Notably, resting CD4+ T cells from MG patients secreted higher levels of VEGFA, TNFRSF9, TWEAK, CCL20, HGF, CCL19, TRAIL, IL18, and TNF-β whereas resting CD8+ T cells secreted higher levels of IL-12B, TRAIL, CCL23, CD244, CXCL11, CCL20, VEGFA, PD-L1, and OSM relative to HC. In contrast, activated CD4+ and CD8+ cells from MG patients exhibited a blunted secretion profile compared to HC, suggesting functional exhaustion. Furthermore, MG-ADL scores correlated with the secretion levels of 14 proteins from resting CD4+ cells, including seven cytokines, five chemokines, and two matrix metalloproteins. Some of the CD4+ T cell secreted proteins also correlated with their corresponding serum or plasma levels *in vivo*. Overall, these findings indicate that T cells in MG exhibit a skewed inflammatory profile characterized by heightened basal activation and impaired inducibility, suggestive of an exhausted phenotype. The interplay between these altered T cell functions and aberrant B cell responses in MG warrants further investigation and may provide novel insights into disease immunopathophysiology as well as opportunities for targeted immunomodulatory therapies.

## 1 Introduction

Myasthenia gravis (MG) is a prototypic autoimmune disorder of the neuromuscular junction, caused by pathogenic autoantibodies that impair signal transmission between motor neurons and skeletal muscle. In most patients (~85%), these antibodies, secreted by autoreactive B cells, target acetylcholine receptors (AChRs) on the postsynaptic membrane. These antibodies cause inhibition of AChR signaling, receptor internalization, complement-mediated damage, and ultimately, fatigable skeletal muscle weakness. In patients who are seronegative for AChR antibodies, antibodies may target other antigens such as the muscle-specific tyrosine kinase (MuSK; ~5-10%) and low-density lipoprotein receptor-related protein 4 (LRP4; ~5-8%), reflecting the heterogeneity of the disease (1). Approximately 5-10% of MG patients are seronegative for those antibodies (2).

Beyond humoral mechanisms, T cells play a pivotal role in orchestrating the autoimmune response in MG (3). Abnormal T cell activation, with dysfunctional T regulatory cells (4), and pathogenic T-B cell interactions contribute to the development and persistence of autoimmunity. The thymus plays a crucial role in eliminating auto-reactive T cells and driving T cell activation, particularly in early-onset MG (EOMG), where thymic hyperplasia and ectopic germinal centers facilitate pathological immune activation. Despite this central role, functional characterization of T cells in MG remains limited, and most studies to date have focused on quantifying immune cell subsets by flow cytometry (5) rather than probing their effector function or inflammatory status. One study reported a twofold higher number of downregulated genes in monocytes from MG patients, suggesting a globally reduced biological activity in these cells (6).

Therapeutic advances over the past decades have led to improved disease control for many patients (7). Immunosuppressants such as corticosteroids and azathioprine (8), B-cell depleting therapies like rituximab, complement inhibitors, including eculizumab and ravulizumab, Fc receptor (FcRn) blockers such as efgartigimod, and thymectomy, all form part of the modern therapeutic arsenal (9). Yet, the exact mechanisms by which these therapies modulate autoimmune dysfunction in human MG remain incompletely understood. Furthermore, circulating antibody titers often fail to correlate with clinical disease severity or treatment response. Currently, disease severity is monitored using clinical scales such as MG Activities of Daily Living (MG-ADL), MG Composite (MGC), and Quantitative MG (QMG), which, while clinically useful, do not reflect real-time autoimmune dynamics in MG patients. Altogether, this highlights the need for more robust and mechanistically informative biomarkers.

Recent studies have begun to reveal alterations in peripheral immune cell populations and serum proteins in MG patients. In AChR antibody seropositive (AChR+) MG, elevated production of IL21, IL4, and also IL-17A has been observed in total CD4+ T cells, suggesting a skewed T cell response (5, 10, 11). Additionally, a recent proteomics analysis of serum identified several differentially expressed proteins, some of which, correlated with disease severity and immunosuppressive treatment status (12). These findings emphasize the potential of immune profiling to support precision medicine approaches in MG. However, the functional state of T cells, particularly their capacity to initiate or sustain autoimmunity, remains incompletely understood.

In this study, we performed in-depth proteomic profiling of resting and activated CD4+ and CD8+ T cells from MG patients and healthy individuals to assess their secretion of inflammatory proteins. Our findings indicate that resting T cells in MG patients exhibit a heightened inflammatory phenotype upon activation, indicating signs of functional exhaustion, suggesting a complex immunopathological role that may influence both disease course and treatment response.

## 2 Materials and methods

### 2.1 MG patients

MG patients with recent disease onset were recruited from the Department of Neurology, Uppsala University Hospital, Uppsala. Age- and sex-matched healthy control (HC) donors were recruited from the Department of Transfusion Medicine at the same hospital. All MG patients had a confirmed ICD-10 diagnosis of G70.0. Abnormal neuromuscular transmission was verified by repetitive nerve stimulation (RNS) and/or single-fiber electromyography (SFEMG), and the majority also had tested positive for AChR antibodies using radioimmunoassay. Clinical records of all MG patients were reviewed to extract information on age at disease onset, MG clinical subgroup, and disease severity, electrophysiological findings, and current and prior treatments (**Table 1**). Clinical subgroups were identified as EOMG, LOMG, or VLOMG (**Table 1**). Disease severity was evaluated at the time of blood sampling using the MG-ADL scale.

**Table 1:** Demographics characteristics of the healthy controls and MG patients.

Variable	HC (N=23)	MG (N=16)	First visit (N=8)	Follow-up visits (N=11)
Age (years)	69 (47 – 71)	70 (55 – 78)		
Sex: M	15 (65.2%)	11 (68.8%)		
AChR+	0 (–)	12 (75%)		
Onset: Early	0 (–)	3 (18.7%)		
Late	0 (–)	6 (37.5%)		
Very late	0 (–)	7 (43.8%)		
Disease duration (years)	0 (–)	1.0 (0.5 - 6.8)		
MG-ADL median (range)	N/A	2 (0–10)	1.5 (0–10)	2 (0–7)
IS: Yes	0 (–)	9 (56.2%)	3 (37.5%)	11 (100%)
Type of IS: Newer biologicals	0 (–)	3 (33.3% of IS)	1 (33.3% of IS)	3 (27.3% of IS)
Thymectomy	0 (–)	1 (6.2%)		

Data are presented as numbers (percentage) or median (Inter Quartile Range, IQR, 25-75% percentile). AChR+, Acetylcholine receptor antibody positive; IS, immunosuppression. MG-ADL, Myasthenia Gravis Activities of Daily Living.

### 2.2 Serum, plasma, and T cell isolation

Venous blood was collected in EDTA-coated tubes for plasma and peripheral blood T cell isolation, and in additive-free tubes for serum collection. Serum tubes were left at room temperature for 30–60 minutes to allow clot formation. Both serum and plasma samples were centrifuged at 4°C at 2200 x g for 10 minutes, aliquoted under cold conditions, and stored at -80°C for further analysis.

Peripheral blood mononuclear cells (PBMCs) were isolated from freshly collected EDTA blood with Ficoll-Paque™ PLUS density gradient media (Cytiva, 17-1440-02) by centrifugation. From PBMCs, T cells were isolated using human CD4+ and CD8+ T cell isolation kit (Milteny Biotec, 130-096–533 and 130-096-495) according to the manufacturer´s protocol and as previously described (13). Cell viability, assessed by trypan blue staining, exceeded 97%. Purified T cells were resuspended in Human Plasma-Like Medium (HPLM; Gibco, A4899101) at a concentration of 1 million per ml and used for further experimentation.

### 2.3 T cell activation

Both CD4+ and CD8+ T cells from MG patients and HC were subjected to antibody-mediated TCR-activation. Cells were seeded (0.1 million cells/well/0.2 ml HPLM) and activated in 96-well plates pre-coated wells with anti-CD3 (5ug/ml; BD Biosciences, 555329) and anti-CD28 (1ug/ml; BD Biosciences, 555726). The cells added in non-coated wells served as resting cells. Cell supernatants were harvested after 72 h of incubation at 37°C, spun down, and saved at -80°C for further analysis with multiplex PEA.

### 2.4 Multiplex PEA

A total of 9 serum, 9 plasma, and 125 cell secreted supernatant samples were analyzed using the Olink^®^ Target 96 Inflammation panel (Olink Proteomics AB, Uppsala, Sweden), which quantifies 92 human inflammation-related proteins including cytokines, chemokines, soluble ligands and their receptors, enzymes (**Supplementary Table 1**). The cohort included MG patients, and age- and sex-matched HC. Samples were randomized in a double-blinded manner and distributed evenly across two 96-well plates to ensure balanced group representation. Proteomic analyses were performed at the Clinical Biomarkers Facility, Science for Life Laboratory, Uppsala University, without disclosing sample identity or donor information to the laboratory. None of the sample failed in quality control.

The proximity extension assay (PEA) (13, 14) was conducted according to the manufacturer’s protocol. Briefly, samples or negative controls were incubated with probe solutions containing 92 antibody pairs, each conjugated to a unique single-stranded DNA oligonucleotide. Upon dual antibody binding to a target protein, the DNA probes come into proximity, hybridize, and are enzymatically extended to form a unique double-stranded DNA reporter. This product is subsequently amplified and quantified using high-throughput microfluidic real-time PCR.

Cycle threshold (Ct) values were normalized to internal spike-in controls, yielding Normalized Protein Expression (NPX) values on a log2 scale. These spike-in controls also served as internal quality metrics for both sample and assay performance. NPX values were generated for all 92 proteins across all samples. NPX is a relative, unitless measure that reflects protein abundance but does not correspond to absolute concentrations. While the assay’s lower limit of detection (LOD) was defined as three standard deviations above background, NPX values below the LOD were retained in the analysis, as low-abundance proteins may still have biological relevance in disease-specific contexts.

### 2.5 MTT assay

After collecting cell supernatants, cellular metabolic activity was determined using MTT (3-(4,5-Dimethylthiazol-2-yl)-2,5-Diphenyl-tetrazolium Bromide) assay (13). Briefly, water-soluble MTT dye with a final concentration of 1 mM was added to each well and cells were incubated for 4 h. Cells were centrifuged to sediment insoluble purple formazan crystals, which were further dissolved in DMSO. Absorbances (optical densities, OD) were measured within10 min using CLARIOStar Plus (BMG Labtech) plate reader at 550nm and a control wavelength 800nm. Cellular metabolic index is calculated as OD_550nm_ – OD_800nm_.

### 2.6 Statistical analysis

The data presentation and statistical analyses were performed on the NPX values, using GraphPad Prism 10.5 and R 4.5.0. Boruta algorithm analysis was performed using Boruta R package to identify linear as well as non-linear relationships. The Boruta algorithm is a wrapper method based on Random Forest that further refines feature selection by introducing shadow features, i.e., the permuted copies of the original biomarkers. The importance of each real biomarker is compared to the maximum importance among its shadow counterparts. Features that consistently exhibit higher importance than the shadows are designated as “confirmed,” whereas less informative features are “rejected.” The Boruta plots were generated using normalized permutation importance, and biomarkers were ranked by the mean of these values across iterations. The algorithm was run for 200 iterations or until all features were definitively classified.

Furthermore, the obtained p-values were corrected for multiple comparisons by using the Benjamini-Hochberg false discovery rate method as per the formula α(i/m). Spearman’s rank correlation test was used to determine any correlation between the protein levels and clinical parameters of the patients, and the strength of correlation was defined based on the Spearman R coefficient, as strong (R= 0.70 - 1.00), or moderate (R= 0.40 - 0.69), or weak (R= 0.10 - 0.39).

## 3 Results

### 3.1 Study cohort and clinical characteristics

This study included 16 MG patients (11 males, 68.8%) with a median age of 70.0 years (IQR 55.0-78.0 years), and 23 age- and sex-matched HCs (15 males, 65.2%) with a median age of 69.0 years (IQR 47.0-71.0 years) (**Table 1**). The MG cohort comprised three EOMG, six LOMG, and seven VLOMG patients. The median disease duration was 1.0 year (IQR: 0.5-6.8 years), with a total 10 patients being newly diagnosed. AChR antibodies were detected in 12 of 16 MG patients. Disease activity as measured by MG-ADL ranged from 0 to 10 (**Table 1**). None of the patients had a thymoma on CT scan, and one patient had been thymectomized. Immunosuppressive treatment was ongoing in nine patients (56.2%), including three who had received rituximab (CD20+ B cell inhibitor) in combination with other immunosuppressive therapies.

### 3.2 T cells from MG patients display an altered secretion profile

To assess the inflammatory secretion profile of T cells, we analyzed cell supernatants using the Olink® Target 96 inflammation panel, which quantifies 92 proteins by using multiplex PEA assay technology, The proteins include several cytokines, chemokines, soluble ligands and their receptors, enzymes, and other proteins connected to inflammatory pathways, and known as biomarkers of inflammation (**Supplementary Table 1**).

#### 3.2.1 CD4+ T cells

**Figure 1:**
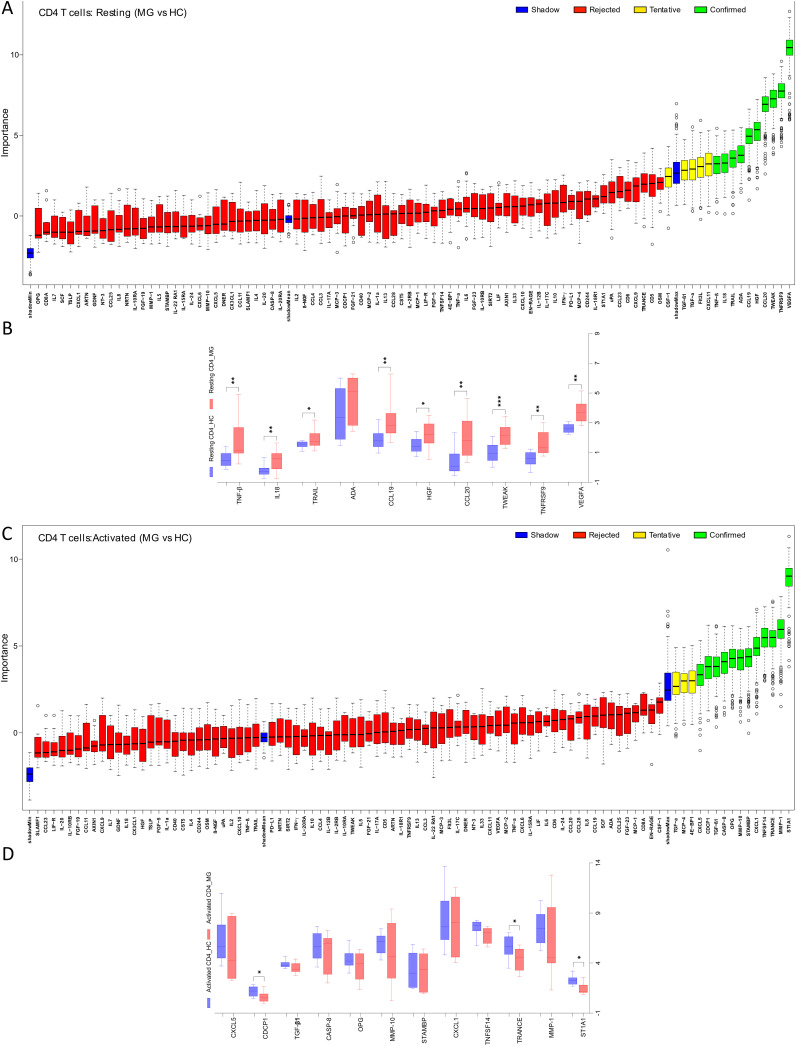
Differential secretion of inflammatory proteins by the CD4+ T cells from MG patients. Boruta algorithm plot showing confirmed (in green), tentative (in yellow), rejected (in red) proteins classifying MG and HC groups for **(A)** resting, and **(C)** activated CD4+ T cells. The confirmed proteins in Boruta analysis were tested with unpaired t-test corrected by Benjamini and Hochberg FDR correction (**Supplementary Table 2**) for **(B)** resting, **(D)** activated CD4+ T cells. Data plotted are NPX values. *p < 0.05, **p < 0.01, ***p < 0.001.

The Boruta algorithm analysis identified 10 proteins that were differentially secreted in the supernatants of resting CD4+ T cell derived from MG patients compared to HC. These included VEGFA, TNFRSF9, TWEAK, CCL20, HGF, CCL19, ADA, TRAIL, IL18, and TNF-β, ranked by decreasing median importance value (**Figure 1A**). Unpaired t-tests with Benjamini-Hochberg correction confirmed significantly higher levels of 9 of these proteins in the MG group, with the exception of ADA (**Figure 1B**, **Supplementary Table 2**). In activated CD4+ T cells, Boruta analyses identified 12 altered proteins in the MG group (**Figure 1C**), whereas an unpaired t-test with Benjamini-Hochberg correction identified only three proteins with reduced secretion, ST1A1, TRANCE, and CDCP1 (**Figure 1D**, **Supplementary Table 2**).

#### 3.2.2 CD8+ T cells

**Figure 2:**
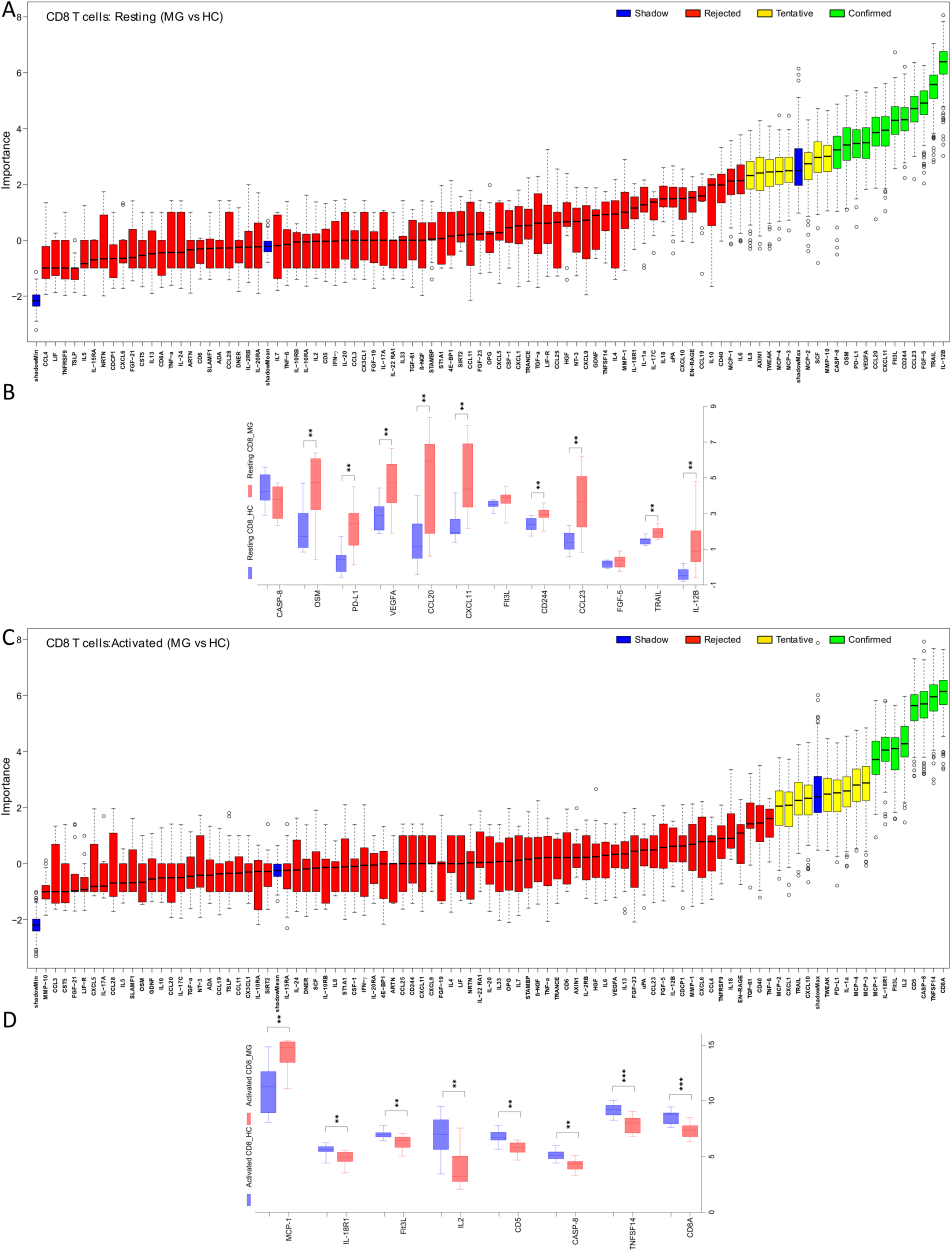
Differential secretion of inflammatory proteins by the CD8+ T cells from MG patients. Boruta algorithm plot showing confirmed (in green), tentative (in yellow), rejected (in red) proteins classifying MG and HC groups for **(A)** resting, and **(C)** activated CD8+ T cells. The confirmed proteins in Boruta analysis were tested with unpaired t-test corrected by Benjamini and Hochberg FDR correction (**Supplementary Table 2**) for **(B)** resting, **(D)** activated CD8+ T cells. Data plotted are NPX values. **p < 0.01, ***p < 0.001.

Analysis of resting CD8+ T cell supernatants with the Boruta algorithm revealed 12 proteins differentially secreted by the cells derived from MG patients (**Figure 2A**). Of these, 9 proteins, IL-12B, TRAIL, CCL23, CD244, CXCL11, CCL20, VEGFA, PD-L1, and OSM, were significantly elevated in the MG group (**Figure 2B**, **Supplementary Table 2**). In activated CD8+ T cells, both Boruta and t-test analyses detected 8 proteins with significantly altered secretion (**Figures 2C, D**). Seven proteins, CD8A, TNFSF14, CASP-8, CD5, IL2, Flt3L, IL-18R1, were significantly reduced in the MG group, whereas MCP-1 was increased (**Figure 2D**, **Supplementary Table 2**).

#### 3.2.3 Activation-dependent changes

**Figure 3:**
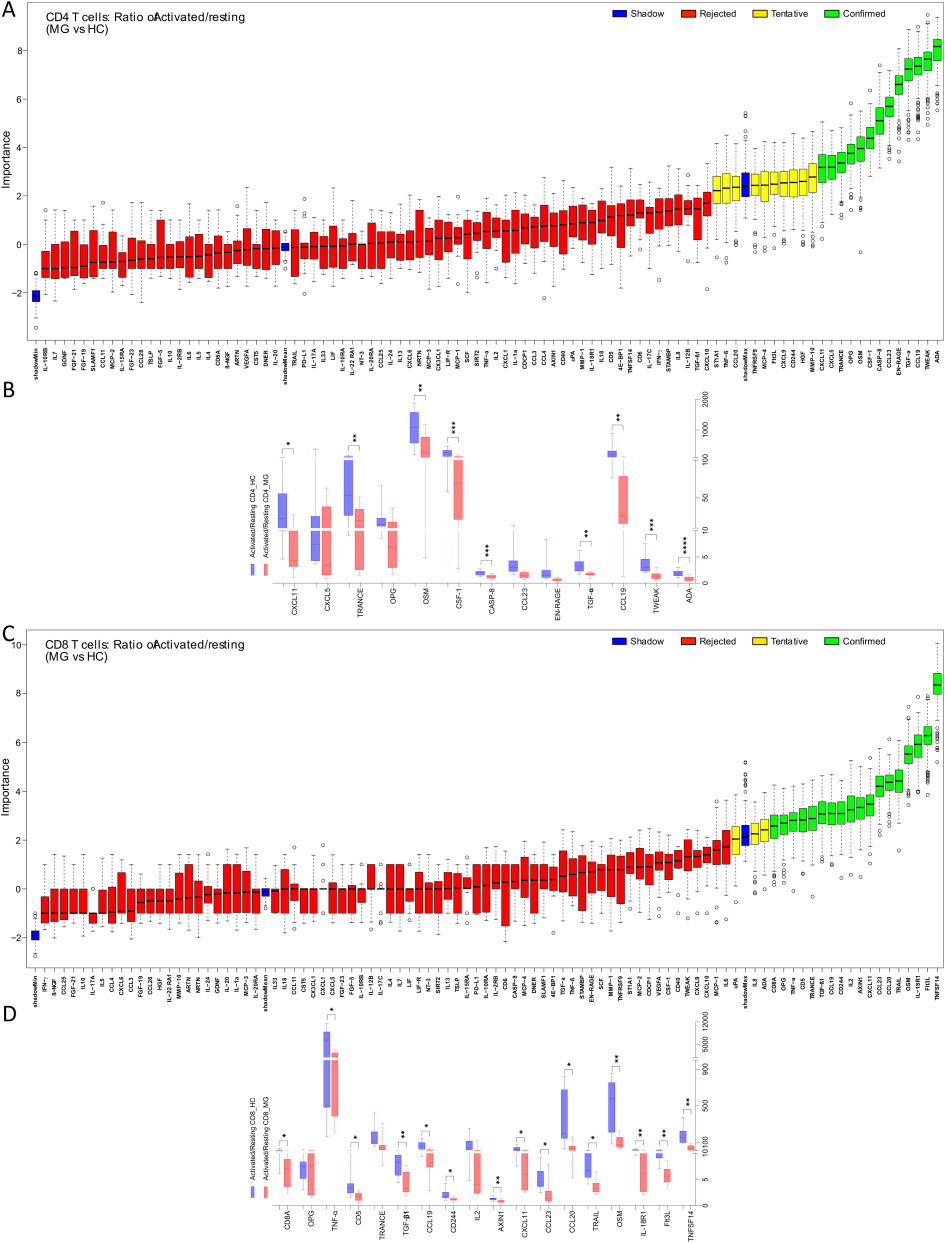
Imbalance of inflammatory protein secretions from CD4+ and CD8+ T cells of MG patients in resting and activated state. The ratio (activated/resting) represents change in protein secretion on activation normalized to the levels at resting state. Boruta algorithm plot shows confirmed proteins, in green, classifying MG and HC groups for **(A)** CD4+, **(C)** CD8+ T cells. The confirmed proteins were tested with unpaired t-test corrected by Benjamini and Hochberg FDR correction (**Supplementary Table 2**) for **(B)** CD4+, **(D)** CD8+ T cells. *p < 0.05, **p < 0.01, ***p < 0.001.

As expected, T cell activation through TCR and co-stimulation induced widespread changes in cytokine and chemokine secretions, as reflected in comparisons between resting and activated states in both CD4+ and CD8+ T cells derived from HC (**Supplementary Figures 1A–C**). To assess disease-specific effects on activation responses in T cells from MG patient, we analyzed fold changes in protein secretion upon activation compared to the resting state (**Figure 3**).

For CD4+ T cells, the Boruta analyses identified 13 activation-associated proteins with altered fold changes, of which 9 proteins, ADA, TWEAK, CCL19, TGF-α, CASP-8, CSF-1, OSM, TRANCE, and CXCL11, were significantly reduced in the MG group (**Figures 3A, B**, **Supplementary Table 2**). Similarly, in CD8+ T cells, 18 proteins were identified by Boruta analyses, and 15 were confirmed to be significantly decreased in MG group. These included TNFSF14, Flt3L, IL-18R1, OSM, TRAIL, CCL20, CCL23, CXCL11, AXIN1, CD244, CCL19, TGF-β1, CD5, TNF-α, and CD8A, (**Figures 3C, D**, **Supplementary Table 2**).

**Figure 4:**
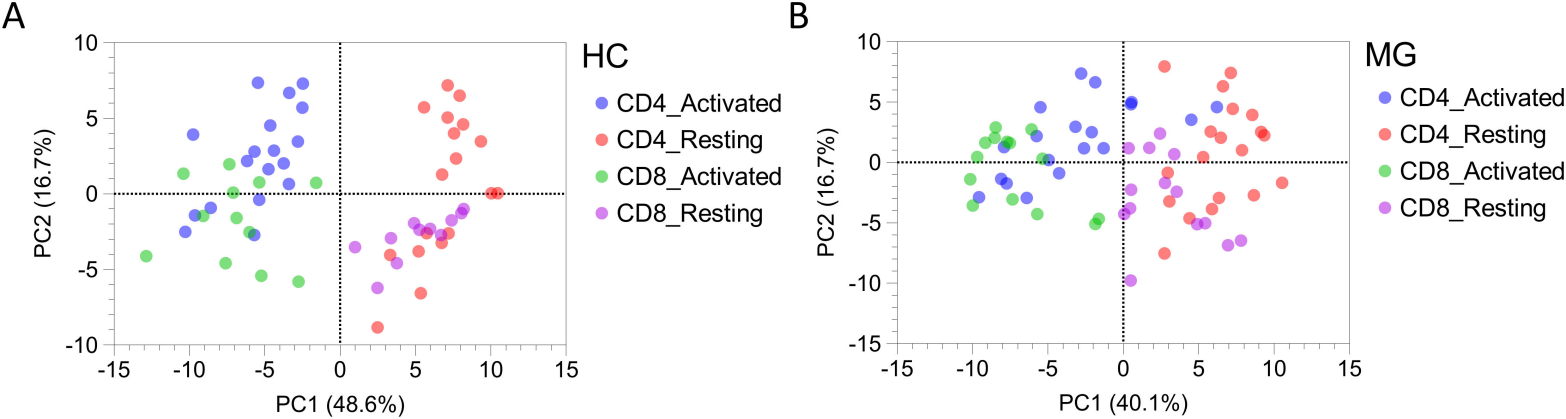
The secretion profiles of T cells derived from MG patients and HCs differs. Principal component analysis (PCA) plot for all 92 proteins secreted by resting and activated CD4+ and CD8+ T cells derived from **(A)** MG patients, and **(B)** HCs.

Principal component analysis (PCA) of all 92 secreted proteins revealed a clear separation between the four T cell subsets (resting and activated CD4+ and CD8+ T cells) in HC (**Figure 4A**). In contrast, this separation was markedly diminished in MG patients, indicating a disruption of normal T cell subset–specific secretion profiles (**Figure 4B**).

### 3.3 The MG-ADL scores correlate with T cell derived protein secretion in MG patients

**Figure 5:**
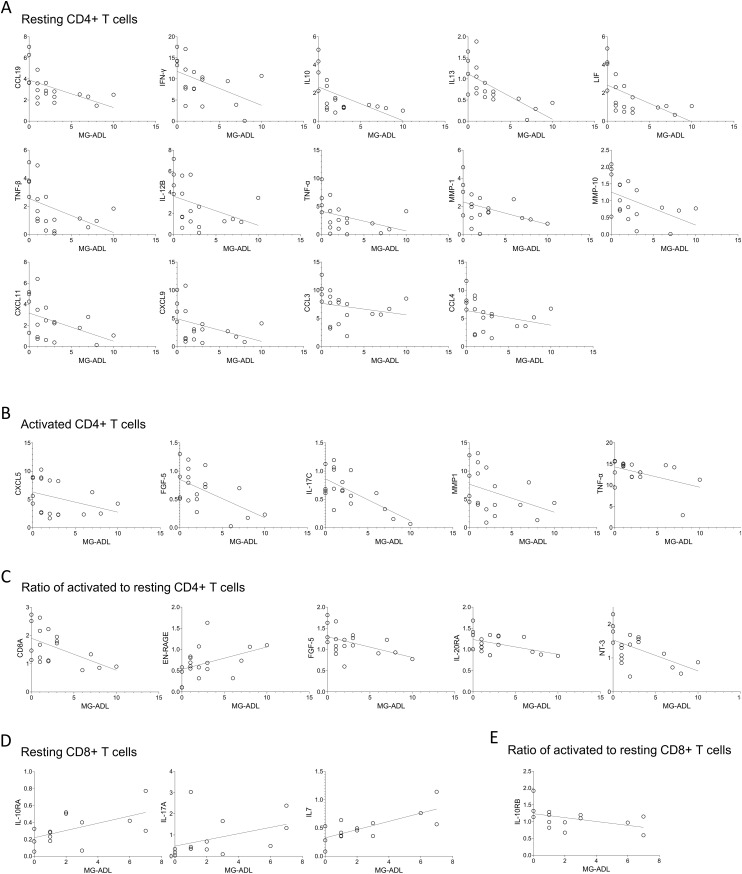
The MG-ADL scores correlate with expression levels of proteins secreted by T cells derived from MG patients. Spearman correlation analysis of MG-ADL scores with the secreted protein levels from **(A)** resting, **(B)** activated, **(C)** ratio of activated to resting CD4+ T cells, **(D)** Resting, and **(E)** ratio of activated to resting CD8+ T cells. Only statistically significant correlations are shown, * p < 0.05. **Supplementary Table 3**, for CD4+ T cells, and **Supplementary Table 4**, for CD8+ T cells, summarizes correlation (spearman R, 95% confidence interval and p-values) of all 92 proteins with the MG-ADL scores.

Spearman correlation analysis revealed significant moderate correlations (Spearman R ranging from 0.40 to 0.69) between MG disease severity, as measured by the MG-ADL score, and the levels of multiple proteins secreted by T cells from MG patients (**Figure 5**). Most correlations were observed in resting CD4+ T cells. Higher MG-ADL scores were associated with lower secretion of several inflammatory and regulatory proteins, including CCL19, IFN-γ, IL10, IL13, LIF, TNF-β, IL-12B, TNF-α, MMP-1, MMP-10, CXCL11, CXCL9, CCL3, and CCL4 (**Figure 5A**, **Supplementary Table 3**).

In activated CD4+ T cells, a similar negative correlation with MG-ADL was found for CXCL5, FGF-5, IL-17C, MMP1, and TNF-α (**Figure 5B**). Additionally, the fold change in CD8A, FGF-5, IL-20RA, and NT-3 upon activation (relative to the resting state) negatively correlated with MG-ADL scores, whereas EN-RAGE had a positive correlation (**Figure 5C**).

Fewer correlations were observed in resting CD8+ T cells, where MG-ADL scores positively correlated with IL-10RA, IL-17A, and IL7 levels. Among activation-induced changes, only IL-10RB fold change showed an inverse correlation with MG-ADL (**Figures 5D, E**, **Supplementary Table 4**).

### 3.4 Specific proteins correlate across serum, plasma and CD4+ T cell supernatant of MG patients

**Figure 6:**
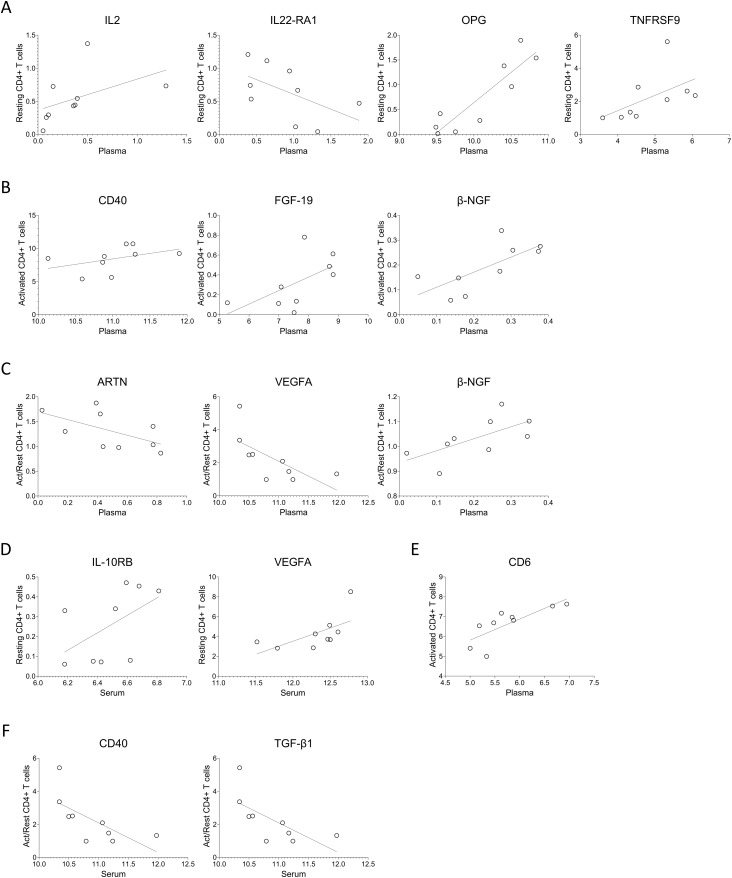
Protein levels in serum or plasma of MG patients correlate with intra-individual protein levels secreted by T cells. **(A–C)** Spearman correlation analysis of the plasma protein levels with that of from **(A)** resting, **(B)** activated, **(C)** ratio of activated to resting CD4+ T cells. **(D–F)** Spearman correlation analysis of the serum protein levels with that of from **(D)** resting, **(E)** activated, **(F)** ratio of activated to resting CD4+ T cells. **Supplementary Tables 5** and **6** summarizes spearman R, 95% confidence interval and p-values for the protein with significant correlations with plasma and serum, respectively.

For a subset of nine MG patients, matched samples of serum, plasma, and CD4+ T cell supernatants were available and analyzed. Spearman correlation analysis revealed significant moderate cross-compartment associations for several proteins. Between plasma and resting CD4+ T cell secretions, IL2, OPG, and TNFRSF9 levels showed positive correlations, while IL-22RA1 levels correlated negatively (**Figure 6A**, **Supplementary Table 5**). In activated CD4+ T cells, CD40, FGF-19, and β-NGF levels positively correlated with plasma protein levels (**Figure 6B**). When comparing plasma with activation-induced fold changes in CD4+ T cells, ARTN and VEGFA correlated negatively, and β-NGF correlated positively (**Figure 6C**).

Serum protein levels also indicated associations with CD4+ T cell secretions. Specifically, IL-10RB and VEGFA showed positive correlations with resting CD4+ T cells, CD6 correlated positively with activated CD4+ T cells, and CD40 and TGF-β1 correlated inversely with the fold change upon activation (**Figures 6D–F**, **Supplementary Table 6**).

### 3.5 T cell secretion profiles differ between first and follow-up visits in MG patients

**Figure 7:**
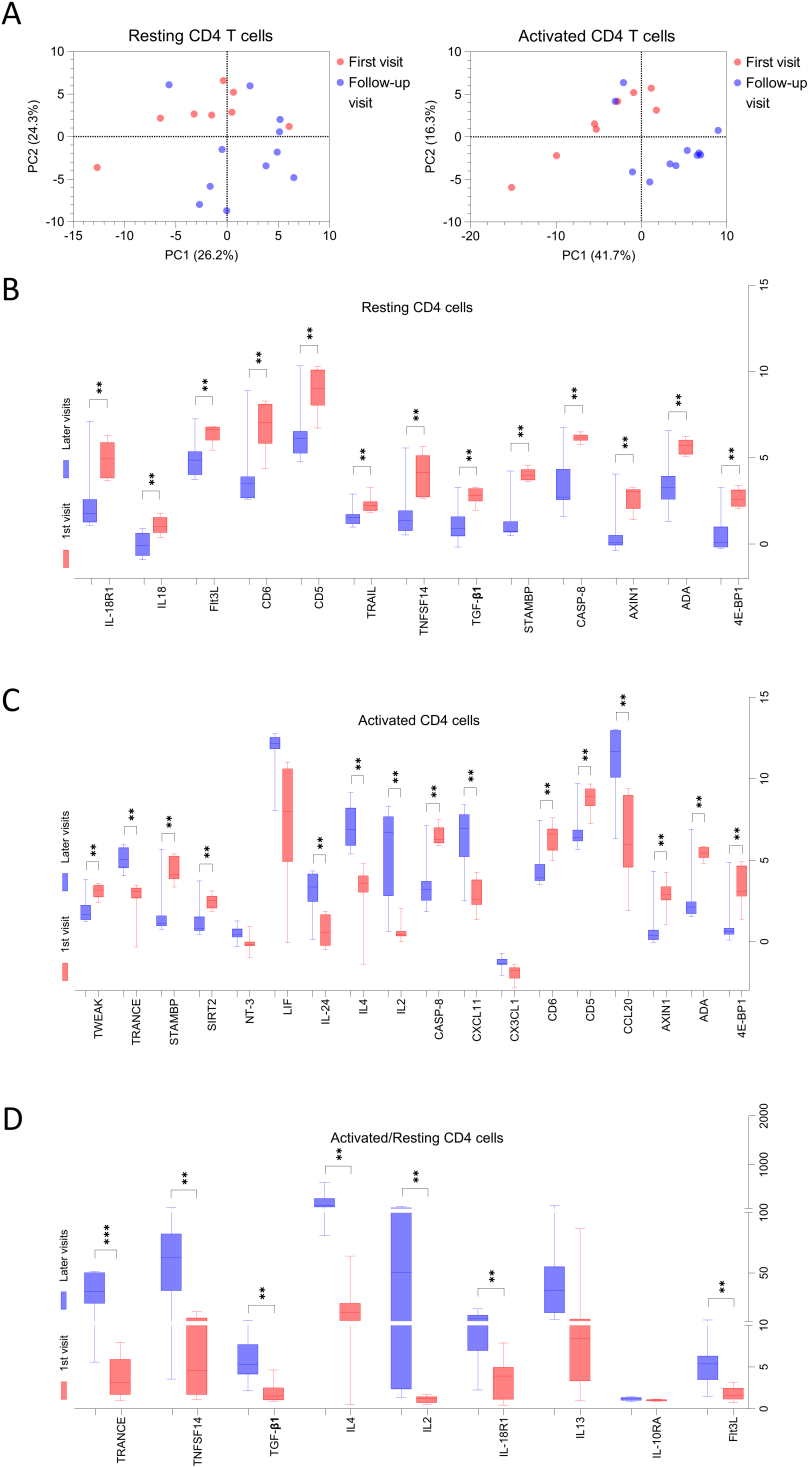
The secretion profiles of T cells derived from MG patients at first visit is different than follow-up visits. **(A)** Principal component analysis (PCA) for all 92 proteins secreted by resting and activated CD4+ T cells derived from MG patients, red dots represent individual patients at 1st visit and blue dots represent at follow-up visits. **(B–D)** The proteins confirmed in Boruta analysis of all 92 proteins for MG patients at first visit versus follow-up visits are shown. Benjamini and Hochberg FDR corrected p-values (**Supplementary Table 7**) are indicated for significantly different proteins for **(B)** resting, **(C)** activated, **(D)** ratio of activated to resting CD4+ T cells. **p < 0.01, ***p < 0.001.

Among the MG patients included in this analysis of CD4+ T cells, eight were newly diagnosed at the time of blood sampling, referred to here as “first visits”, while the remaining samples were obtained during follow-up visits approximately every three months (**Table 1**). PCA of the 92 proteins secreted by resting and activated CD4+ T cells revealed a partial separation between samples collected at the first versus follow-up visits, suggesting a shift in T cell secretory profiles over time or with treatment (**Figure 7A**).

Subsequent analysis using the Boruta algorithm (data not shown), followed by unpaired t-tests with Benjamini-Hochberg FDR correction, identified significant differences in the secretion levels of several proteins across the visits (**Figures 7B–D**, **Supplementary Table 7**), indicating dynamic changes in T cell function during the course of disease and treatment.

In resting CD4+ T cell supernatants, 13 proteins were significantly elevated at first visit compared to follow-up. These included 4E-BP1, ADA, AXIN1, CASP-8, STAMBP, TGF-β1, TNFSF14, TRAIL, CD5, CD6, Flt3L, IL18, and IL-18R1 (**Figure 7B**). In contrast, supernatants from activated CD4+ T cells, at the first visit exhibited higher levels of 4E-BP1, ADA, AXIN1, CD5, CD6, CASP-8, SIRT2, STAMBP, TWEAK and lower levels of CCL20, CXCL11, IL2, IL4, IL24, and TRANCE (**Figure 7C**). Finally, analysis of fold changes between activated and resting conditions revealed a significantly reduced induction of Flt3L, IL-18R1, IL2, IL4, TGF-β1, TNFSF14, and TRANCE in MG patients at their first visit compared to follow-up (**Figure 7D**).

### 3.6 T cells from MG patients are metabolically hyperactive

**Figure 8:**
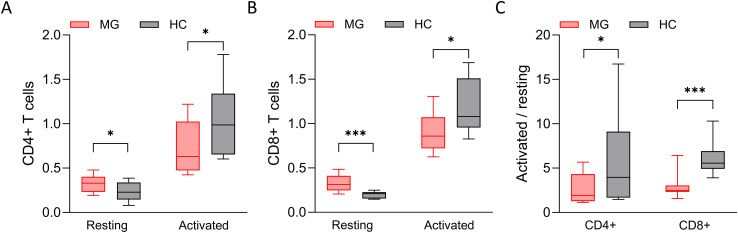
The cellular metabolic index of T cells. The cellular metabolic index, measured by MTT assay, of **(A)** resting and activated CD4+ T cells derived from MG patients and HCs, and of **(B)** CD8+ T cells. **(C)** The ratio of cellular metabolic index of activated and resting T cells. n = 16 MG patients and 16 HCs for CD4+ T cells, and n = 14 MG patients and 11 HCs for CD8+ T cells. *p < 0.05, ***p < 0.001.

Given the distinct secretion profiles observed in CD4+ and CD8+ T cells from MG patients compared to HCs, we assessed the cellular metabolic activity using an MTT assay as an indirect measure of proliferation (**Figure 8**). In the resting state, both T cell subsets from MG patients showed slightly elevated metabolic activity compared to age- and sex-matched HCs (**Figures 8A, B**). However, following activation, MG-derived T cells did not reach to the same metabolic levels as those from HCs (**Figures 8A, B**). The fold-change analysis suggests a blunt metabolic response in MG-derived T cells, indicative of functional exhaustion (**Figure 8C**). These differences were more prominent in CD8+ T cells, a relatively understudied cell population in MG, highlighting their potential relevance in future studies.

## 4 Discussion

This study provides novel insights into the functional phenotype of circulating CD4+ and CD8+ T cells in MG. Our findings indicate that these T cells from MG patients display an intrinsically pro-inflammatory profile, characterized by increased secretion of inflammatory proteins. Upon antibody-mediated T cell receptor (TCR) stimulation, however, these cells exhibit an attenuated activation response compared to HC. This dual pattern, suggestive of chronic pre-activation and functional exhaustion, is consistent with prior observations of chronic immune activation and altered T cell subset distribution in MG, particularly among CD4+ effector and regulatory T cells (10, 15). Importantly, levels of certain inflammatory proteins secreted by MG T cells *in vitro* correlated with clinical disease severity, as assessed by the MG-ADL score. Moreover, a set of proteins in cell supernatants also reflected the patients’ *in vivo* inflammation milieu, showing concordance with their native levels in serum and plasma. Together, these findings support the notion that T cell-derived inflammatory signals may contribute both to disease pathogenesis and symptom burden in MG.

Resting CD4+ T cell from MG patients exhibited a differential secretion profile than HC, consistent with a hyperactive and pro-inflammatory state. Elevated secretion levels of autoimmune mediators, including VEGFA, TNFRSF9, TWEAK, TRAIL, and chemokines such as CCL20, suggest a hyperactive CD4+ T cell state associated with chronic inflammation in MG. VEGFA plays a role in thymocyte maturation and migration into the peripheral circulation (16), and is also involved in the regeneration of neuromuscular junctions following nerve injury (17). TNFRSF9, a co-stimulatory receptor on effector and memory T cells, is typically upregulated following strong T cell activation (18–20) and promotes clonal expansion, proliferation, and the development of Th1 cell responses. It is also expressed by follicular dendritic cells and contributes to B cell activation within germinal centers of the thymus (21). TWEAK is a pro-inflammatory cytokine capable of inducing skeletal muscle atrophy under denervated conditions. It also possesses chemotactic properties and may facilitate T cell infiltration at the neuromuscular junction (22, 23). Elevated levels of chemokines such as CCL20 and CCL19 may reflect enhanced recruitment of Th17 cells and dendritic cells to inflammatory sites (24–27). Notably, increased frequencies of circulating CCR6+ Th17 cells (responsive to chemotactic signals of CCL20) cells have been reported in MG (26, 27), while data on CCR7+ dendritic cells (responsive to CCL19) are conflicting (28, 29). TWEAK (TNFSF12) and TRAIL (TNFSF10), both linked to inflammation-induced apoptosis and tissue damage (30, 31), were also elevated, further supporting a pro-inflammatory milieu. Moreover, plasma TNFRSF9 and serum VEGFA levels correlated positively with their secretion from CD4+ T cells, implicating these cells as a major source. Interestingly, despite higher secretion levels in MG, CCL19, and TNF-β showed moderate negative correlations with MG-ADL scores, suggesting a potential regulatory role in limiting disease severity.

Resting CD8+ T cells from MG patients exhibited elevated secretion of several inflammatory and regulatory proteins, including IL-12B, TRAIL, CCL23, CD244, CXCL11, CCL20, VEGFA, PD-L1, and OSM, compared to HC. This profile suggests a potential shift in CD8+ T cell activation toward a chronically activated and possibly functionally exhausted phenotype, characteristic of maladaptive immune responses during persistent inflammation. Notably, CD244, and PD-L1 are markers associated with sustained T cell activation, which may lead to progressive loss of effector function (10, 32, 33). In line with our findings, increased frequencies of circulating activated PD-L1+ T cells have been previously been reported in MG patients (10, 11), further supporting a state of chronic immune stimulation. Several of the upregulated chemokines, including CCL23, CXCL11, and CCL20, are well-characterized for their role in recruiting monocytes, T cells, NK cells, dendritic cells, and macrophages to sites of inflammation (24, 34). Alongside chemokines, IL-12B and TRAIL, both linked to cytotoxic T cell responses and apoptotic signaling, likely contribute to sustained immune activation at key anatomical sites such as the neuromuscular junction and the thymus (35, 36). Of note, CCL20, VEGFA, and TRAIL were secreted at higher levels by both resting CD4+ and CD8+ T cells in MG patients, suggesting shared pathways of T cell dysregulation that may underlie systemic inflammation in the disease. In support of this, studies in a murine model of systemic lupus have shown that both CD4+ and CD8+ T cells secrete approximately twice as much soluble TNFRSF9 with CD8+ T cells also expressing higher levels of the surface receptor (37). Importantly, soluble TNFRSF9 levels in that context correlate with T cell activation and cell death, but not proliferation (37), suggesting a role in chronic immune stimulation and functional exhaustion. In human MG, elevated expression of TRAIL receptors DR4 and DR5 has been observed in the thymus, highlighting a potential role for TRAIL in shaping thymic T cell composition and central tolerance mechanisms (38). Furthermore, IL-12B (also known as IL-12p40) has been linked to genetic susceptibility, increased expression, and a pro-inflammatory role in other autoimmune diseases such as psoriasis (36) and multiple sclerosis (39, 40), further supporting its contribution to the inflammatory milieu in MG.

In contrast to the findings in resting T cells, T cells from MG patients exhibited a markedly attenuated response to stimulation. Upon activation, CD4+ T cells secreted lower levels of ST1A1, TRANCE, and CDCP1, while CD8+ T cells secreted reduced amounts of CD8A, TNFSF14, CASP-8, CD5, IL2, Flt3L, IL-18R1 compared to HC. This hyporesponsive profile may reflect diminished cytotoxic potential, particularly due to reduced CD8A and CASP-8 (41, 42), which are critical for target cell elimination, as well as impaired T cell-APCs interactions and co-stimulatory signaling, associated with lower levels of Flt3L, and TNFSF14. Together, these alterations may contribute to dysfunctional immune regulation and a failure to resolve inflammation, thereby facilitating chronic disease progression in MG.

As a consequence of dysregulated T cell function in MG, several proteins showed altered fold changes between resting and activated states. The top 5 candidates differentially regulated in CD4+ T cells were ADA, TWEAK, CCL19, TGF-α, and CASP-8, and in CD8+ T cells were TNFSF14, Flt3L, IL-18R1, OSM, and TRAIL. Notably, CCL19 and CXCL11 were consistently altered in both subsets. CCL19 and CXCL11 signal through CCR7 and CXCR3 receptors, respectively, and are both secreted by thymic endothelial cells surrounding the medullary vessels. CCR7 is expressed on CD4+ and CD8+ T cells as well as dendritic cells within the thymus. Activation of the CCR7-CCL19 and CXCL11-CXCR3 signaling pathways facilitates the chemotactic migration of CD4+ and CD8+ T cells into the thymic medulla, supporting their positive selection and maturation (43). Some proteins, including VEGFA, CCL20, CXCL11, and HGF, were elevated in both CD4+ and CD8+ cells and in the serum of the corresponding patients, suggesting T cells as a key source. Conversely, TNF-β, and OSM were increased in T cells but lower in serum, pointing to compartment-specific regulation (5, 12). Additionally, both T cell subtypes from MG patients exhibited higher metabolic activity at rest but reduced activity upon activation. Together, these findings indicate a hyperactive yet functionally exhausted phenotype in both CD4+ and CD8+ T cells in MG, reminiscent of the T cell dysregulation observed in hyper-inflammatory conditions like myasthenic crisis (44) and COVID-19 (45). Single cell transcriptomic analysis of TCR repertoires in PBMCs from MG patients experiencing myasthenic crisis revealed a persistently activated state and clonal expansion of several T cell subsets, including CD4+ and CD8+ cells, consistent with features of T cell exhaustion (44).

A comparable study using the Olink T96 Inflammation panel examined cytokine secretion profiles from PBMCs of patients with type 1 diabetes (T1D) and non-diabetic controls. Upon activation with anti-CD3 antibody, T1D-derived PBMSs showed slightly lower levels of TNFRSF9, CXCL9, TWEAK, CXCL11 and higher levels of IL-2RB and SCF proteins in culture supernatants (13). These findings contrast with those observed in our MG cohort, despite both MG and T1D being autoimmune disorders. In multiple sclerosis (MS), CD4+ T cells secrete elevated levels of IL2, IL4, IL5, IL10, IL13, IL17, IL21, IL22, TNF-α, IFN-γ (46, 47), primarily measured by ELISA. The secretion profile observed in our study may be specific to MG; however, further direct comparisons with other neurological autoimmune diseases using the same experimental conditions are needed to confirm disease specificity.

One aim of the study was to understand the effect of immunosuppressive treatments on the differences in T cell inflammatory profiles in newly diagnosed patients at their first visit versus follow-up visits in the clinic. The primary differences between the visits were a three-month interval and initiation or change in immunosuppressive treatment with either prednisone, azathioprine, rituximab or combination of two of these. PCA of both resting and activated CD4+ T cells revealed partial separation between first and follow-up visits, suggesting temporal changes in secreted protein profiles in both resting and activated state. Specifically, 13 proteins were elevated in resting CD4+ T cells at the first visit, while 9 were elevated and 6 reduced in the activated state at the first visit compared to follow-up. These findings suggest that T cells from newly diagnosed MG patients have a more pronounced inflammatory profile compared to a more attenuated profile over time due to treatment and clinical factors. These results also indicate that changes in immunosuppressive treatment status can directly influence the activation profile, and dynamic immunomodulation of CD4^+^ T cells, thereby their proteomic signatures secreted in circulation. Immunosuppression may therefore act as a confounding factor when evaluating biomarkers related to disease progression, treatment response, or disease severity. It should be strictly accounted for in both preclinical biomarker discovery and clinical trials.

The study has some limitations. The cross-sectional design and modest size limited subgroup analyses, particularly between immunosuppression treatment-naïve versus immunosuppressed MG patients. T cell secretion profiles may be influenced by ongoing immunosuppression and may not fully capture disease-intrinsic alterations. While our findings highlight increased basal secretion and attenuated responses upon activation, further validation using longitudinal cohorts and complementary methods (e.g., qPCR, flow cytometry for T cell exhaustion and hyperactivity markers like LAG-3, TIGIT, PD-L1) would strengthen mechanistic insights. Functional assays (e.g., cytotoxicity, degranulation chemotaxis) may also enhance interpretation. Additionally, although the Boruta algorithm provides a robust framework for biomarker discovery, its rankings carry some degree of uncertainty. Despite these limitations, the study offers important proteomic evidence of immune dysregulation in MG and lays a foundation for future biomarker-driven research. Including other autoimmune disease controls in future studies will be critical to confirm disease specificity.

In conclusion, these findings indicate that T cells in MG display a dysregulated inflammatory profile, characterized by heightened basal activation and an exhausted phenotype. The interplay between dysfunctional T cell activity and aberrant B cell responses merits further investigation to clarify their functional roles in MG pathogenesis and treatment response. Together, our findings highlight T cells as potential contributors to disease progression and as targets for future immunomodulatory therapies.

## Data Availability

The original contributions presented in the study are included in the article/**Supplementary Material**. Further inquiries can be directed to the corresponding authors.
