# Supplementary material for: Inflammatory imbalance and activation deficits in T cells of myasthenia gravis patients revealed by proteomic profiling
Source: Front Immunol. 2025 Aug 4;16:1648020. doi: 10.3389/fimmu.2025.1648020 (PMC12358477; doi:10.3389/fimmu.2025.1648020)
Supplement: Supplementary file 1 [file DataSheet1.pdf]

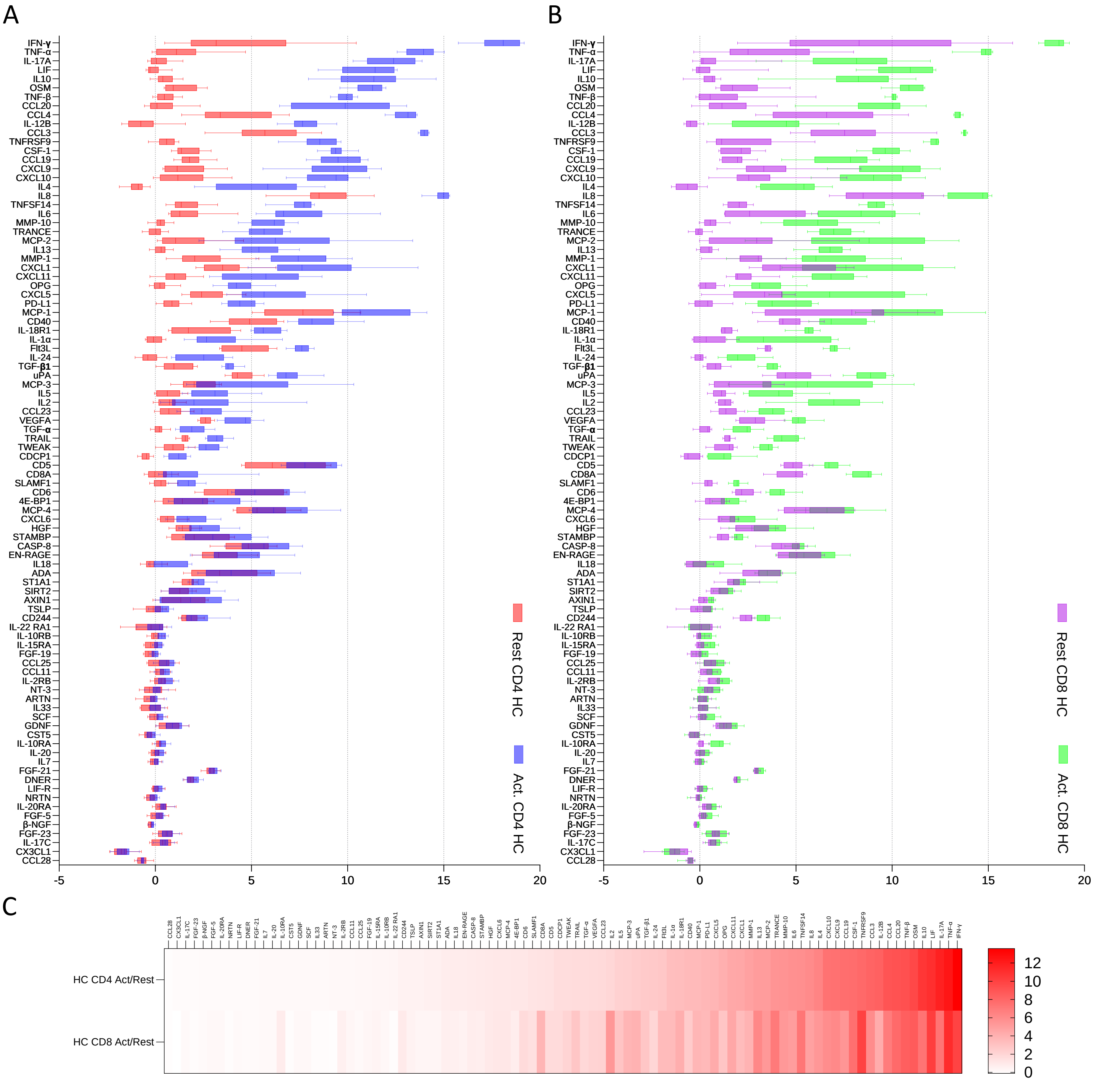


**Supplementary Figure 1: A profile of inflammatory proteins secreted by T cells from healthy controls upon activation.** Box-whiskers plot of 92 proteins in the inflammation panel from resting (red) and activated (blue) CD4+ T cells **(A)** and resting (magenta) and activated (green) CD8+ T cells **(B)**. Data plotted as NPX values. **(C)** Heatmap showing the relative abundance (median fold change) of the secreted proteins upon activation compared to the resting state in CD4+ and CD8+ T cells.

## Supplementary Table 1. Olink's Inflammation Panel, Protein assay list (Product number: 95302)

| | | | |
|---|---|---|---|
| Adenosine Deaminase (ADA) | P00813 | Fibroblast growth factor 23 (FGF-23) | Q9GZV9 |
| Artemin (ARTN) | Q5T4W7 | Fibroblast growth factor 5 (FGF-5) | Q8NF90 |
| Axin-1 (AXIN1) | O15169 | Fibroblast growth factor 19 (FGF-19) | O95750 |
| Beta-nerve growth factor (β-NGF) | P01138 | Fms-related tyrosine kinase 3 ligand (Flt3L) | P49771 |
| Caspase-8 (CASP-8) | Q14790 | Fractalkine (CX3CL1) | P78423 |
| C-C motif chemokine 3 (CCL3) | P10147 | Glial cell line-derived neurotrophic factor (GDNF) | P39905 |
| C-C motif chemokine 4 (CCL4) | P13236 | Hepatocyte growth factor (HGF) | P14210 |
| C-C motif chemokine 19 (CCL19) | Q99731 | Interferon gamma (IFN-γ) | P01579 |
| C-C motif chemokine 20 (CCL20) | P78556 | Interleukin-1 alpha (IL-1A) | P01583 |
| C-C motif chemokine 23 (CCL23) | P55773 | Interleukin-2 (IL-2) | P60568 |
| C-C motif chemokine 25 (CCL25) | O15444 | Interleukin-2 receptor subunit beta (IL-2RB) | P14784 |
| C-C motif chemokine 28 (CCL28) | Q9NRJ3 | Interleukin-4 (IL-4) | P05112 |
| CD40L receptor (CD40) | P25942 | Interleukin-5 (IL5) | P05113 |
| CUB domain-containing protein 1 (CDCP1) | Q9H5V8 | Interleukin-6 (IL6) | P05231 |
| C-X-C motif chemokine 1 (CXCL1) | P09341 | Interleukin-7 (IL-7) | P13232 |
| C-X-C motif chemokine 5 (CXCL5) | P42830 | Interleukin-8 (IL-8) | P10145 |
| C-X-C motif chemokine 6 (CXCL6) | P80162 | Interleukin-10 (IL10) | P22301 |
| C-X-C motif chemokine 9 (CXCL9) | Q07325 | Interleukin-10 receptor subunit alpha (IL-10RA) | Q13651 |
| C-X-C motif chemokine 10 (CXCL10) | P02778 | Interleukin-10 receptor subunit beta (IL-10RB) | Q08334 |
| C-X-C motif chemokine 11 (CXCL11) | O14625 | Interleukin-12 subunit beta (IL-12B) | P29460 |
| Cystatin D (CST5) | P28325 | Interleukin-13 (IL-13) | P35225 |
| Delta and Notch-like epidermal growth factor-related receptor (DNER) | Q8NFT8 | Interleukin-15 receptor subunit alpha (IL-15RA) | Q13261 |
| Eotaxin (CCL11) | P51671 | Interleukin-17A (IL-17A) | Q16552 |
| Eukaryotic translation initiation factor 4E-binding protein 1 (4E-BP1) | Q13541 | Interleukin-17C (IL-17C) | Q9P0M4 |
| Fibroblast growth factor 21 (FGF-21) | Q9NSA1 | Interleukin-18 (IL-18) | Q14116 |
| Interleukin-18 receptor 1 (IL-18R1) | Q13478 | Programmed cell death 1 ligand 1 (PD-L1) | Q9NZQ7 |
| Interleukin-20 (IL-20) | Q9NYY1 | Protein S100-A12 (EN-RAGE) | P80511 |
| Interleukin-20 receptor subunit alpha (IL-20RA) | Q9UHF4 | Signaling lymphocytic activation molecule (SLAMF1) | Q13291 |
| Interleukin-22 receptor subunit alpha-1 (IL-22 RA1) | Q8N6P7 | SIR2-like protein 2 (SIRT2) | Q8IXJ6 |
| Interleukin-24 (IL-24) | Q13007 | STAM-binding protein (STAMBP) | O95630 |
| Interleukin-33 (IL-33) | O95760 | Stem cell factor (SCF) | P21583 |
| Latency-associated peptide transforming growth factor β1 (TGF-β1) | P01137 | Sulfotransferase 1A1 (ST1A1) | P50225 |
| Leukemia inhibitory factor (LIF) | P15018 | T cell surface glycoprotein CD6 isoform (CD6) | Q8WWJ7 |
| Leukemia inhibitory factor receptor (LIF-R) | P42702 | T-cell surface glycoprotein CD5 (CD5) | P06127 |
| Macrophage colony-stimulating factor 1 (CSF-1) | P09603 | T-cell surface glycoprotein CD8 alpha chain (CD8A) | P01732 |
| Matrix metalloproteinase-1 (MMP-1) | P03956 | Thymic stromal lymphopoietin (TSLP) | Q969D9 |
| Matrix metalloproteinase-10 (MMP-10) | P09238 | TNF-beta (TNF-β) | P01374 |
| Monocyte chemotactic protein 1 (MCP-1) | P13500 | TNF-related activation-induced cytokine (TRANCE) | O14788 |
| Monocyte chemotactic protein 2 (MCP-2) | P80075 | TNF-related apoptosis-inducing ligand (TRAIL) | P50591 |
| Monocyte chemotactic protein 3 (MCP-3) | P80098 | Transforming growth factor alpha (TGF-α) | P01135 |
| Monocyte chemotactic protein 4 (MCP-4) | Q99616 | Tumor necrosis factor (Ligand) superfamily, member 12 (TWEAK) | O43508 |
| Natural killer cell receptor 2B4 (CD244) | Q9BZW8 | Tumor necrosis factor alpha (TNF-α) | P01375 |
| Neurotrophin-3 (NT-3) | P20783 | Tumor necrosis factor ligand superfamily member 14 (TNFSF14) | O43557 |
| Neurturin (NRTN) | Q99748 | Tumor necrosis factor receptor superfamily member 9 (TNFRSF9) | Q07011 |
| Oncostatin-M (OSM) | P13725 | Urokinase-type plasminogen activator (uPA) | P00749 |
| Osteoprotegerin (OPG) | O00300 | Vascular endothelial growth factor A (VEGF-A) | P15692 |

**Supplementary Table 2: Secreted proteins from T cells significantly differed in MG patients compared to HCs.**

| Resting CD4+ T cells (MG vs HC) | | |
|---|---|---|
| | P value | FDR q Value |
| VEGFA | 0.0036 | 0.0060 |
| TNFRSF9 | 0.0006 | 0.0030 |
| TWEAK | 0.0001 | 0.0010 |
| CCL20 | 0.0010 | 0.0032 |
| HGF | 0.0139 | 0.0173 |
| CCL19 | 0.0016 | 0.0040 |
| ADA | 0.1662 | 0.1662 |
| TRAIL | 0.0251 | 0.0279 |
| IL18 | 0.0047 | 0.0067 |
| TNF-β | 0.0031 | 0.0060 |

| Resting CD8+ T cells (MG vs HC) | | |
|---|---|---|
| | P value | FDR q Value |
| IL-12B | 0.0036 | 0.0054 |
| TRAIL | 0.0004 | 0.0017 |
| FGF-5 | 0.2385 | 0.2385 |
| CCL23 | 0.0008 | 0.0019 |
| CD244 | 0.0029 | 0.0052 |
| Flt3L | 0.1448 | 0.1580 |
| CXCL11 | 0.0003 | 0.0017 |
| CCL20 | 0.0006 | 0.0017 |
| VEGFA | 0.0030 | 0.0052 |
| PD-L1 | 0.0005 | 0.0017 |
| OSM | 0.0047 | 0.0062 |
| CASP-8 | 0.0827 | 0.0993 |

| Activated CD4+ T cells (MG vs HC) | | |
|---|---|---|
| | P value | FDR q Value |
| ST1A1 | 0.0020 | 0.0160 |
| MMP-1 | 0.2510 | 0.3012 |
| TRANCE | 0.0027 | 0.0160 |
| TNFSF14 | 0.0171 | 0.0512 |
| CXCL1 | 0.5551 | 0.6020 |
| STAMBP | 0.6020 | 0.6020 |
| MMP-10 | 0.2202 | 0.2972 |
| OPG | 0.0617 | 0.1233 |
| CASP-8 | 0.1557 | 0.2668 |
| TGF-β1 | 0.0245 | 0.0588 |
| CDCP1 | 0.0100 | 0.0399 |
| CXCL5 | 0.2229 | 0.2972 |

| Activated CD8+ T cells (MG vs HC) | | |
|---|---|---|
| | P value | FDR q Value |
| CD8A | 0.0001 | 0.0004 |
| TNFSF14 | 0.0001 | 0.0004 |
| CASP-8 | 0.0005 | 0.0013 |
| CD5 | 0.0009 | 0.0018 |
| IL2 | 0.0022 | 0.0029 |
| Flt3L | 0.0015 | 0.0023 |
| IL-18R1 | 0.0040 | 0.0040 |
| MCP-1 | 0.0026 | 0.0030 |

| Activated/Resting CD4+ T cells (MG vs HC) | | |
|---|---|---|
| | P value | FDR q Value |
| ADA | <0.000001 | 0.0000 |
| TWEAK | 0.0001 | 0.0005 |
| CCL19 | 0.0056 | 0.0090 |
| TGF-α | 0.0016 | 0.0036 |
| EN-RAGE | 0.1910 | 0.1910 |
| CCL23 | 0.1534 | 0.1813 |
| CASP-8 | 0.0001 | 0.0005 |
| CSF-1 | 0.0002 | 0.0005 |
| OSM | 0.0014 | 0.0036 |
| OPG | 0.0818 | 0.1063 |
| TRANCE | 0.0026 | 0.0048 |
| CXCL5 | 0.1743 | 0.1889 |
| CXCL11 | 0.0075 | 0.0108 |

| Activated/Resting CD8+ T cells (MG vs HC) | | |
|---|---|---|
| | P value | FDR q Value |
| TNFSF14 | 0.0014 | 0.0062 |
| Flt3L | 0.0033 | 0.0098 |
| IL-18R1 | 0.0002 | 0.0023 |
| OSM | 0.0007 | 0.0042 |
| TRAIL | 0.0056 | 0.0132 |
| CCL20 | 0.0062 | 0.0132 |
| CCL23 | 0.0105 | 0.0153 |
| CXCL11 | 0.0081 | 0.0146 |
| AXIN1 | 0.0022 | 0.0078 |
| IL2 | 0.0681 | 0.0766 |
| CD244 | 0.0110 | 0.0153 |
| CCL19 | 0.0066 | 0.0132 |
| TGF-β1 | 0.0003 | 0.0023 |
| TRANCE | 0.1054 | 0.1116 |
| CD5 | 0.0388 | 0.0466 |
| TNF-α | 0.0223 | 0.0287 |
| OPG | 0.5555 | 0.5555 |
| CD8A | 0.0101 | 0.0153 |

Proteins confirmed in Boruta algorithm are varified by unpaired t test with Welch correction and false discovery rate method by Benjamini and Hochberg. n = 19 MG patients, 18 HCs for CD4+ T cells, and n = 14 MG patients, 11 HCs for CD8+ T cells.

**Supplementary Table 3: Correlation of MG-ADL scores of MG patients with all 92 proteins secreted from CD4+ T cells.**

| MG-ADL vs | Resting CD4+ T cells | | | | Activated CD4+ T cells | | | | Ratio of activated to resting CD4+ T cells | | | |
|---|---|---|---|---|---|---|---|---|---|---|---|---|
| | Spearman r | 95% CI | P value | Signi. | Spearman r | 95% CI | P value | Signi. | Spearman r | 95% CI | P value | Signi. |
| 4E-BP1 | 0.117 | -0.3688 to 0.5524 | 0.634 | ns | 0.119 | -0.3673 to 0.5537 | 0.629 | ns | -0.104 | -0.5430 to 0.3805 | 0.673 | ns |
| ADA | 0.283 | -0.2105 to 0.6614 | 0.241 | ns | 0.278 | -0.2152 to 0.6586 | 0.249 | ns | 0.194 | -0.2990 to 0.6047 | 0.427 | ns |
| ARTN | 0.263 | -0.2307 to 0.6493 | 0.276 | ns | -0.210 | -0.6152 to 0.2837 | 0.389 | ns | -0.432 | -0.7472 to 0.04232 | 0.065 | ns |
| AXIN1 | 0.021 | -0.4494 to 0.4815 | 0.934 | ns | 0.193 | -0.2998 to 0.6041 | 0.429 | ns | 0.077 | -0.4033 to 0.5237 | 0.755 | ns |
| CASP-8 | 0.048 | -0.4270 to 0.5025 | 0.845 | ns | 0.065 | -0.4130 to 0.5151 | 0.791 | ns | -0.013 | -0.4760 to 0.4551 | 0.957 | ns |
| CCL11 | -0.012 | -0.4754 to 0.4558 | 0.960 | ns | -0.059 | -0.5105 to 0.4182 | 0.811 | ns | -0.225 | -0.6250 to 0.2690 | 0.355 | ns |
| CCL19 | -0.691 | -0.8749 to -0.3315 | 0.001 | ** | -0.264 | -0.6498 to 0.2298 | 0.275 | ns | 0.235 | -0.2593 to 0.6313 | 0.334 | ns |
| CCL20 | -0.342 | -0.6965 to 0.1474 | 0.152 | ns | -0.359 | -0.7063 to 0.1284 | 0.132 | ns | -0.137 | -0.5668 to 0.3507 | 0.575 | ns |
| CCL23 | -0.284 | -0.6619 to 0.2096 | 0.239 | ns | -0.199 | -0.6082 to 0.2939 | 0.414 | ns | 0.224 | -0.2698 to 0.6245 | 0.357 | ns |
| CCL25 | 0.373 | -0.1122 to 0.7145 | 0.116 | ns | -0.155 | -0.5790 to 0.3346 | 0.526 | ns | -0.443 | -0.7530 to 0.02910 | 0.058 | ns |
| CCL28 | 0.053 | -0.4234 to 0.5059 | 0.831 | ns | 0.136 | -0.3523 to 0.5655 | 0.580 | ns | -0.139 | -0.5680 to 0.3491 | 0.570 | ns |
| CCL3 | -0.476 | -0.7705 to -0.01272 | 0.040 | * | 0.051 | -0.4248 to 0.5045 | 0.836 | ns | 0.330 | -0.1601 to 0.6897 | 0.168 | ns |
| CCL4 | -0.464 | -0.7644 to 0.00215 | 0.045 | * | -0.323 | -0.6855 to 0.1679 | 0.177 | ns | 0.223 | -0.2707 to 0.6239 | 0.359 | ns |
| CD244 | -0.360 | -0.7068 to 0.1274 | 0.131 | ns | 0.204 | -0.2888 to 0.6117 | 0.402 | ns | 0.259 | -0.2352 to 0.6465 | 0.285 | ns |
| CD40 | -0.269 | -0.6526 to 0.2252 | 0.266 | ns | 0.099 | -0.3843 to 0.5398 | 0.687 | ns | 0.253 | -0.2406 to 0.6431 | 0.295 | ns |
| CD5 | 0.044 | -0.4307 to 0.4992 | 0.859 | ns | 0.377 | -0.1070 to 0.7170 | 0.111 | ns | 0.323 | -0.1679 to 0.6855 | 0.177 | ns |
| CD6 | 0.060 | -0.4175 to 0.5112 | 0.808 | ns | 0.362 | -0.1244 to 0.7084 | 0.128 | ns | 0.299 | -0.1937 to 0.6711 | 0.214 | ns |
| CD8A | 0.204 | -0.2888 to 0.6117 | 0.402 | ns | -0.305 | -0.6749 to 0.1871 | 0.204 | ns | -0.483 | -0.7743 to -0.02198 | 0.036 | * |
| CDCP1 | 0.164 | -0.3264 to 0.5851 | 0.502 | ns | -0.165 | -0.5857 to 0.3256 | 0.500 | ns | -0.160 | -0.5820 to 0.3305 | 0.514 | ns |
| CSF-1 | -0.448 | -0.7559 to 0.02243 | 0.055 | ns | -0.431 | -0.7467 to 0.04341 | 0.066 | ns | 0.091 | -0.3912 to 0.5340 | 0.711 | ns |
| CST5 | 0.182 | -0.3099 to 0.5970 | 0.456 | ns | -0.101 | -0.5410 to 0.3828 | 0.681 | ns | -0.287 | -0.6641 to 0.2059 | 0.233 | ns |
| CX3CL1 | -0.256 | -0.6448 to 0.2379 | 0.290 | ns | -0.049 | -0.5032 to 0.4263 | 0.842 | ns | 0.250 | -0.2442 to 0.6409 | 0.302 | ns |
| CXCL1 | -0.062 | -0.5125 to 0.4160 | 0.802 | ns | -0.161 | -0.5827 to 0.3297 | 0.511 | ns | -0.187 | -0.6006 to 0.3049 | 0.442 | ns |
| CXCL10 | -0.393 | -0.7256 to 0.08941 | 0.096 | ns | -0.360 | -0.7068 to 0.1274 | 0.131 | ns | 0.200 | -0.2931 to 0.6088 | 0.412 | ns |
| CXCL11 | -0.473 | -0.7691 to -0.00927 | 0.041 | * | -0.220 | -0.6222 to 0.2733 | 0.365 | ns | 0.170 | -0.3207 to 0.5893 | 0.486 | ns |
| CXCL5 | -0.293 | -0.6673 to 0.2003 | 0.224 | ns | -0.502 | -0.7845 to -0.04787 | 0.028 | * | -0.304 | -0.6743 to 0.1880 | 0.205 | ns |
| CXCL6 | -0.148 | -0.5741 to 0.3410 | 0.545 | ns | -0.080 | -0.5263 to 0.4003 | 0.744 | ns | -0.068 | -0.5171 to 0.4108 | 0.783 | ns |
| CXCL9 | -0.493 | -0.7794 to -0.03485 | 0.032 | * | -0.393 | -0.7256 to 0.08941 | 0.096 | ns | 0.253 | -0.2406 to 0.6431 | 0.295 | ns |
| DNER | -0.095 | -0.5366 to 0.3882 | 0.700 | ns | -0.117 | -0.5524 to 0.3688 | 0.634 | ns | 0.071 | -0.4078 to 0.5197 | 0.772 | ns |
| EN-RAGE | 0.189 | -0.3032 to 0.6018 | 0.438 | ns | 0.384 | -0.09982 to 0.7206 | 0.105 | ns | 0.492 | 0.03367 to 0.7789 | 0.033 | * |
| FGF-19 | 0.199 | -0.2939 to 0.6082 | 0.414 | ns | -0.204 | -0.6117 to 0.2888 | 0.402 | ns | -0.340 | -0.6955 to 0.1493 | 0.155 | ns |
| FGF-21 | -0.212 | -0.6170 to 0.2811 | 0.383 | ns | -0.209 | -0.6147 to 0.2845 | 0.391 | ns | 0.079 | -0.4018 to 0.5250 | 0.749 | ns |
| FGF-23 | 0.152 | -0.3378 to 0.5766 | 0.535 | ns | -0.282 | -0.6608 to 0.2115 | 0.242 | ns | -0.425 | -0.7433 to 0.05104 | 0.070 | ns |
| FGF-5 | 0.132 | -0.3554 to 0.5631 | 0.590 | ns | -0.477 | -0.7715 to -0.01503 | 0.039 | * | -0.476 | -0.7705 to -0.01272 | 0.040 | * |
| Flt3L | 0.079 | -0.4011 to 0.5256 | 0.747 | ns | -0.122 | -0.5562 to 0.3641 | 0.618 | ns | -0.185 | -0.5988 to 0.3074 | 0.449 | ns |
| GDNF | 0.055 | -0.4212 to 0.5079 | 0.822 | ns | -0.307 | -0.6759 to 0.1852 | 0.201 | ns | -0.251 | -0.6415 to 0.2433 | 0.301 | ns |
| HGF | -0.063 | -0.5138 to 0.4145 | 0.797 | ns | 0.000 | -0.4656 to 0.4656 | >0.9999 | ns | -0.176 | -0.5929 to 0.3157 | 0.472 | ns |
| IFN-γ | -0.604 | -0.8348 to -0.1925 | 0.006 | ** | -0.015 | -0.4774 to 0.4537 | 0.951 | ns | 0.381 | -0.1029 to 0.7190 | 0.108 | ns |
| IL10 | -0.685 | -0.8725 to -0.3224 | 0.001 | ** | -0.347 | -0.6996 to 0.1414 | 0.145 | ns | -0.054 | -0.5065 to 0.4226 | 0.828 | ns |
| IL-10RA | 0.072 | -0.4071 to 0.5204 | 0.769 | ns | -0.112 | -0.5487 to 0.3735 | 0.649 | ns | -0.063 | -0.5138 to 0.4145 | 0.797 | ns |
| IL-10RB | 0.238 | -0.2558 to 0.6336 | 0.326 | ns | 0.075 | -0.4048 to 0.5223 | 0.760 | ns | -0.023 | -0.4836 to 0.4473 | 0.925 | ns |
| IL-12B | -0.567 | -0.8167 to -0.1371 | 0.011 | * | -0.183 | -0.5976 to 0.3091 | 0.454 | ns | 0.304 | -0.1880 to 0.6743 | 0.205 | ns |
| IL13 | -0.805 | -0.9241 to -0.5424 | <0.0001 | **** | -0.079 | -0.5256 to 0.4011 | 0.747 | ns | 0.105 | -0.3789 to 0.5442 | 0.668 | ns |
| IL-15RA | 0.076 | -0.4041 to 0.5230 | 0.758 | ns | -0.178 | -0.5941 to 0.3140 | 0.467 | ns | -0.146 | -0.5729 to 0.3427 | 0.550 | ns |
| IL-17A | -0.454 | -0.7592 to 0.01459 | 0.051 | ns | -0.421 | -0.7414 to 0.05538 | 0.073 | ns | -0.118 | -0.5531 to 0.3680 | 0.631 | ns |
| IL-17C | 0.019 | -0.4508 to 0.4802 | 0.939 | ns | -0.591 | -0.8284 to -0.1724 | 0.008 | ** | -0.390 | -0.7241 to 0.09254 | 0.099 | ns |
| IL18 | 0.183 | -0.3091 to 0.5976 | 0.454 | ns | -0.004 | -0.4691 to 0.4621 | 0.986 | ns | -0.095 | -0.5366 to 0.3882 | 0.700 | ns |
| IL-18R1 | 0.029 | -0.4430 to 0.4877 | 0.908 | ns | 0.162 | -0.3281 to 0.5839 | 0.507 | ns | 0.005 | -0.4614 to 0.4698 | 0.983 | ns |
| IL-1α | -0.230 | -0.6285 to 0.2637 | 0.343 | ns | -0.376 | -0.7160 to 0.1091 | 0.113 | ns | -0.409 | -0.7345 to 0.07040 | 0.082 | ns |
| IL2 | 0.063 | -0.4145 to 0.5138 | 0.797 | ns | -0.402 | -0.7310 to 0.07784 | 0.088 | ns | -0.389 | -0.7236 to 0.09359 | 0.100 | ns |
| IL-20 | 0.276 | -0.2179 to 0.6570 | 0.253 | ns | -0.109 | -0.5468 to 0.3758 | 0.657 | ns | -0.163 | -0.5845 to 0.3272 | 0.504 | ns |
| IL-20RA | 0.249 | -0.2451 to 0.6403 | 0.304 | ns | -0.089 | -0.5327 to 0.3927 | 0.716 | ns | -0.457 | -0.7606 to 0.01122 | 0.049 | * |
| IL-22 RA1 | -0.278 | -0.6586 to 0.2152 | 0.249 | ns | 0.068 | -0.4108 to 0.5171 | 0.783 | ns | 0.111 | -0.3743 to 0.5480 | 0.652 | ns |
| IL-24 | -0.172 | -0.5905 to 0.3190 | 0.481 | ns | -0.287 | -0.6641 to 0.2059 | 0.233 | ns | -0.269 | -0.6531 to 0.2243 | 0.265 | ns |
| IL-2RB | -0.113 | -0.5499 to 0.3719 | 0.644 | ns | -0.290 | -0.6657 to 0.2031 | 0.229 | ns | -0.050 | -0.5039 to 0.4256 | 0.839 | ns |
| IL33 | 0.160 | -0.3305 to 0.5820 | 0.514 | ns | -0.158 | -0.5808 to 0.3321 | 0.519 | ns | -0.187 | -0.6000 to 0.3057 | 0.445 | ns |
| IL4 | -0.185 | -0.5988 to 0.3074 | 0.449 | ns | -0.127 | -0.5593 to 0.3602 | 0.605 | ns | -0.058 | -0.5099 to 0.4190 | 0.814 | ns |
| IL5 | 0.143 | -0.3459 to 0.5705 | 0.560 | ns | -0.006 | -0.4705 to 0.4607 | 0.980 | ns | -0.251 | -0.6415 to 0.2433 | 0.301 | ns |
| IL6 | -0.228 | -0.6273 to 0.2655 | 0.347 | ns | -0.330 | -0.6897 to 0.1601 | 0.168 | ns | -0.136 | -0.5655 to 0.3523 | 0.580 | ns |
| IL7 | 0.067 | -0.4115 to 0.5165 | 0.786 | ns | -0.020 | -0.4809 to 0.4501 | 0.936 | ns | 0.009 | -0.4586 to 0.4726 | 0.971 | ns |
| IL8 | -0.377 | -0.7165 to 0.1081 | 0.112 | ns | -0.102 | -0.5417 to 0.3820 | 0.679 | ns | 0.362 | -0.1244 to 0.7084 | 0.128 | ns |

| | | | | | | | | | | | | |
|---|---|---|---|---|---|---|---|---|---|---|---|---|
| LIF | -0.691 | -0.8753 to -0.3330 | 0.001 | ** | -0.327 | -0.6881 to 0.1631 | 0.171 | ns | -0.017 | -0.4788 to 0.4522 | 0.945 | ns |
| LIF-R | 0.015 | -0.4537 to 0.4774 | 0.951 | ns | 0.104 | -0.3805 to 0.5430 | 0.673 | ns | 0.027 | -0.4444 to 0.4863 | 0.913 | ns |
| MCP-1 | -0.346 | -0.6991 to 0.1424 | 0.147 | ns | -0.230 | -0.6285 to 0.2637 | 0.343 | ns | 0.282 | -0.2115 to 0.6608 | 0.242 | ns |
| MCP-2 | -0.455 | -0.7597 to 0.01347 | 0.050 | ns | -0.255 | -0.6443 to 0.2388 | 0.292 | ns | 0.220 | -0.2733 to 0.6222 | 0.365 | ns |
| MCP-3 | -0.449 | -0.7564 to 0.02131 | 0.054 | ns | -0.253 | -0.6426 to 0.2415 | 0.297 | ns | -0.155 | -0.5790 to 0.3346 | 0.526 | ns |
| MCP-4 | -0.291 | -0.6663 to 0.2022 | 0.227 | ns | -0.092 | -0.5346 to 0.3904 | 0.708 | ns | 0.372 | -0.1132 to 0.7140 | 0.117 | ns |
| MMP-1 | -0.504 | -0.7854 to -0.05025 | 0.028 | * | -0.508 | -0.7872 to -0.05503 | 0.027 | * | -0.309 | -0.6770 to 0.1833 | 0.199 | ns |
| MMP-10 | -0.510 | -0.7886 to -0.05863 | 0.026 | * | -0.322 | -0.6850 to 0.1689 | 0.179 | ns | -0.327 | -0.6876 to 0.1640 | 0.172 | ns |
| NRTN | 0.168 | -0.3231 to 0.5875 | 0.493 | ns | -0.284 | -0.6619 to 0.2096 | 0.239 | ns | -0.243 | -0.6364 to 0.2514 | 0.317 | ns |
| NT-3 | 0.076 | -0.4041 to 0.5230 | 0.758 | ns | -0.448 | -0.7559 to 0.02243 | 0.055 | ns | -0.494 | -0.7803 to -0.03720 | 0.032 | * |
| OPG | -0.119 | -0.5537 to 0.3673 | 0.629 | ns | -0.087 | -0.5314 to 0.3942 | 0.722 | ns | -0.131 | -0.5624 to 0.3562 | 0.593 | ns |
| OSM | -0.446 | -0.7549 to 0.02466 | 0.056 | ns | -0.443 | -0.7535 to 0.02799 | 0.057 | ns | 0.164 | -0.3264 to 0.5851 | 0.502 | ns |
| PD-L1 | -0.338 | -0.6944 to 0.1513 | 0.157 | ns | -0.274 | -0.6559 to 0.2198 | 0.257 | ns | 0.016 | -0.4530 to 0.4781 | 0.948 | ns |
| SCF | 0.101 | -0.3828 to 0.5410 | 0.681 | ns | 0.222 | -0.2716 to 0.6233 | 0.361 | ns | 0.095 | -0.3874 to 0.5372 | 0.698 | ns |
| SIRT2 | 0.183 | -0.3091 to 0.5976 | 0.454 | ns | 0.188 | -0.3040 to 0.6012 | 0.440 | ns | -0.051 | -0.5045 to 0.4248 | 0.836 | ns |
| SLAMF1 | 0.050 | -0.4256 to 0.5039 | 0.839 | ns | -0.487 | -0.7766 to -0.02781 | 0.034 | * | -0.392 | -0.7251 to 0.09046 | 0.097 | ns |
| ST1A1 | 0.018 | -0.4515 to 0.4795 | 0.942 | ns | -0.061 | -0.5118 to 0.4167 | 0.805 | ns | -0.127 | -0.5593 to 0.3602 | 0.605 | ns |
| STAMBP | 0.267 | -0.2271 to 0.6515 | 0.270 | ns | 0.135 | -0.3531 to 0.5649 | 0.582 | ns | 0.034 | -0.4387 to 0.4918 | 0.890 | ns |
| TGF-α | -0.206 | -0.6129 to 0.2871 | 0.397 | ns | -0.170 | -0.5887 to 0.3215 | 0.488 | ns | 0.077 | -0.4033 to 0.5237 | 0.755 | ns |
| TGF-β1 | 0.079 | -0.4018 to 0.5250 | 0.749 | ns | 0.204 | -0.2888 to 0.6117 | 0.402 | ns | 0.028 | -0.4437 to 0.4870 | 0.911 | ns |
| TNFRSF9 | -0.367 | -0.7109 to 0.1193 | 0.123 | ns | -0.126 | -0.5587 to 0.3610 | 0.608 | ns | 0.176 | -0.3157 to 0.5929 | 0.472 | ns |
| TNFSF14 | 0.119 | -0.3673 to 0.5537 | 0.629 | ns | -0.061 | -0.5118 to 0.4167 | 0.805 | ns | -0.067 | -0.5165 to 0.4115 | 0.786 | ns |
| TNF-α | -0.544 | -0.8057 to -0.1053 | 0.016 | * | -0.462 | -0.7635 to 0.00443 | 0.046 | * | 0.115 | -0.3704 to 0.5512 | 0.639 | ns |
| TNF-β | -0.619 | -0.8420 to -0.2158 | 0.005 | ** | -0.385 | -0.7216 to 0.09775 | 0.103 | ns | 0.259 | -0.2352 to 0.6465 | 0.285 | ns |
| TRAIL | 0.124 | -0.3626 to 0.5575 | 0.613 | ns | -0.047 | -0.5019 to 0.4278 | 0.848 | ns | 0.010 | -0.4579 to 0.4733 | 0.968 | ns |
| TRANCE | -0.185 | -0.5988 to 0.3074 | 0.449 | ns | -0.120 | -0.5550 to 0.3657 | 0.623 | ns | -0.118 | -0.5531 to 0.3680 | 0.631 | ns |
| TSLP | 0.005 | -0.4614 to 0.4698 | 0.983 | ns | -0.392 | -0.7251 to 0.09046 | 0.097 | ns | -0.287 | -0.6641 to 0.2059 | 0.233 | ns |
| TWEAK | -0.023 | -0.4836 to 0.4473 | 0.925 | ns | 0.052 | -0.4241 to 0.5052 | 0.833 | ns | 0.075 | -0.4048 to 0.5223 | 0.760 | ns |
| uPA | -0.170 | -0.5887 to 0.3215 | 0.488 | ns | 0.156 | -0.3338 to 0.5796 | 0.523 | ns | 0.280 | -0.2133 to 0.6597 | 0.245 | ns |
| VEGFA | 0.342 | -0.1474 to 0.6965 | 0.152 | ns | 0.162 | -0.3289 to 0.5833 | 0.509 | ns | -0.300 | -0.6717 to 0.1928 | 0.212 | ns |
| β-NGF | -0.203 | -0.6112 to 0.2896 | 0.404 | ns | -0.242 | -0.6358 to 0.2522 | 0.319 | ns | -0.173 | -0.5911 to 0.3182 | 0.479 | ns |

# XY Pairs for CD4+ T cells = 19

The correlation was assessed by Spearman correaltion analysis.

**Supplementary Table 4: Correlation of MG-ADL scores of MG patients with all 92 proteins secreted from CD8+ T cells.**

| MG-ADL vs | Resting CD8+ T cells | | | | Activated CD8+ T cells | | | | Ratio of activated to resting CD8+ T cells | | | |
|---|---|---|---|---|---|---|---|---|---|---|---|---|
| | Spearman r | 95% CI | P value | Signi. | Spearman r | 95% CI | P value | Signi. | Spearman r | 95% CI | P value | Signi. |
| 4E-BP1 | 0.063 | -0.4972 to 0.5858 | 0.832 | ns | -0.036 | -0.5678 to 0.5172 | 0.905 | ns | -0.069 | -0.5902 to 0.4921 | 0.814 | ns |
| ADA | -0.054 | -0.5798 to 0.5040 | 0.856 | ns | 0.004 | -0.5398 to 0.5462 | 0.991 | ns | 0.146 | -0.4315 to 0.6382 | 0.617 | ns |
| ARTN | -0.231 | -0.6876 to 0.3570 | 0.424 | ns | -0.101 | -0.6104 to 0.4678 | 0.731 | ns | 0.128 | -0.4463 to 0.6272 | 0.662 | ns |
| AXIN1 | -0.155 | -0.6436 to 0.4240 | 0.595 | ns | -0.045 | -0.5739 to 0.5106 | 0.880 | ns | 0.081 | -0.4835 to 0.5975 | 0.784 | ns |
| CASP-8 | -0.013 | -0.5524 to 0.5335 | 0.966 | ns | 0.004 | -0.5398 to 0.5462 | 0.991 | ns | 0.085 | -0.4801 to 0.6004 | 0.772 | ns |
| CCL11 | 0.282 | -0.3080 to 0.7156 | 0.326 | ns | -0.251 | -0.6987 to 0.3382 | 0.384 | ns | -0.181 | -0.6595 to 0.4011 | 0.532 | ns |
| CCL19 | 0.058 | -0.5006 to 0.5828 | 0.844 | ns | -0.060 | -0.5843 to 0.4989 | 0.838 | ns | -0.459 | -0.8022 to 0.1116 | 0.100 | ns |
| CCL20 | -0.029 | -0.5632 to 0.5222 | 0.923 | ns | -0.134 | -0.6313 to 0.4408 | 0.645 | ns | -0.013 | -0.5524 to 0.5335 | 0.966 | ns |
| CCL23 | 0.022 | -0.5270 to 0.5586 | 0.942 | ns | -0.121 | -0.6231 to 0.4517 | 0.679 | ns | -0.123 | -0.6244 to 0.4499 | 0.673 | ns |
| CCL25 | 0.410 | -0.1712 to 0.7794 | 0.146 | ns | -0.190 | -0.6647 to 0.3933 | 0.511 | ns | -0.372 | -0.7612 to 0.2145 | 0.190 | ns |
| CCL28 | 0.159 | -0.4203 to 0.6462 | 0.584 | ns | 0.056 | -0.5023 to 0.5813 | 0.850 | ns | 0.085 | -0.4801 to 0.6004 | 0.772 | ns |
| CCL3 | -0.121 | -0.6231 to 0.4517 | 0.679 | ns | 0.094 | -0.4731 to 0.6061 | 0.748 | ns | 0.143 | -0.4334 to 0.6368 | 0.623 | ns |
| CCL4 | 0.103 | -0.4661 to 0.6118 | 0.725 | ns | -0.090 | -0.6033 to 0.4766 | 0.760 | ns | -0.072 | -0.5917 to 0.4904 | 0.808 | ns |
| CD244 | -0.282 | -0.7156 to 0.3080 | 0.326 | ns | -0.052 | -0.5784 to 0.5056 | 0.862 | ns | 0.081 | -0.4835 to 0.5975 | 0.784 | ns |
| CD40 | -0.309 | -0.7297 to 0.2811 | 0.280 | ns | -0.117 | -0.6203 to 0.4553 | 0.690 | ns | 0.173 | -0.4088 to 0.6542 | 0.552 | ns |
| CD5 | -0.152 | -0.6422 to 0.4259 | 0.601 | ns | -0.204 | -0.6724 to 0.3814 | 0.481 | ns | 0.125 | -0.4481 to 0.6258 | 0.667 | ns |
| CD6 | -0.047 | -0.5754 to 0.5090 | 0.874 | ns | 0.013 | -0.5335 to 0.5524 | 0.966 | ns | -0.020 | -0.5571 to 0.5286 | 0.948 | ns |
| CD8A | -0.013 | -0.5524 to 0.5335 | 0.966 | ns | 0.013 | -0.5335 to 0.5524 | 0.966 | ns | 0.002 | -0.5414 to 0.5446 | 0.997 | ns |
| CDCP1 | 0.188 | -0.3952 to 0.6634 | 0.516 | ns | -0.141 | -0.6354 to 0.4352 | 0.628 | ns | -0.231 | -0.6876 to 0.3570 | 0.424 | ns |
| CSF-1 | 0.031 | -0.5205 to 0.5648 | 0.917 | ns | -0.206 | -0.6737 to 0.3794 | 0.476 | ns | -0.329 | -0.7400 to 0.2603 | 0.248 | ns |
| CST5 | -0.150 | -0.6409 to 0.4278 | 0.606 | ns | -0.166 | -0.6502 to 0.4146 | 0.568 | ns | -0.128 | -0.6272 to 0.4463 | 0.662 | ns |
| CX3CL1 | 0.047 | -0.5090 to 0.5754 | 0.874 | ns | -0.043 | -0.5723 to 0.5123 | 0.886 | ns | -0.300 | -0.7250 to 0.2901 | 0.295 | ns |
| CXCL1 | -0.018 | -0.5555 to 0.5303 | 0.954 | ns | -0.029 | -0.5632 to 0.5222 | 0.923 | ns | -0.293 | -0.7215 to 0.2969 | 0.306 | ns |
| CXCL10 | -0.246 | -0.6963 to 0.3424 | 0.393 | ns | -0.244 | -0.6950 to 0.3445 | 0.397 | ns | 0.137 | -0.4389 to 0.6327 | 0.639 | ns |
| CXCL11 | -0.123 | -0.6244 to 0.4499 | 0.673 | ns | -0.052 | -0.5784 to 0.5056 | 0.862 | ns | 0.181 | -0.4011 to 0.6595 | 0.532 | ns |
| CXCL5 | -0.146 | -0.6382 to 0.4315 | 0.617 | ns | -0.043 | -0.5723 to 0.5123 | 0.886 | ns | -0.166 | -0.6502 to 0.4146 | 0.568 | ns |
| CXCL6 | -0.125 | -0.6258 to 0.4481 | 0.667 | ns | 0.146 | -0.4315 to 0.6382 | 0.617 | ns | 0.094 | -0.4731 to 0.6061 | 0.748 | ns |
| CXCL9 | -0.166 | -0.6502 to 0.4146 | 0.568 | ns | -0.287 | -0.7180 to 0.3036 | 0.318 | ns | 0.058 | -0.5006 to 0.5828 | 0.844 | ns |
| DNER | -0.253 | -0.6999 to 0.3361 | 0.379 | ns | -0.217 | -0.6801 to 0.3693 | 0.452 | ns | 0.045 | -0.5106 to 0.5739 | 0.880 | ns |
| EN-RAGE | -0.009 | -0.5493 to 0.5367 | 0.979 | ns | 0.078 | -0.4853 to 0.5960 | 0.790 | ns | 0.229 | -0.3591 to 0.6863 | 0.429 | ns |
| FGF-19 | 0.461 | -0.1088 to 0.8032 | 0.098 | ns | 0.134 | -0.4408 to 0.6313 | 0.645 | ns | -0.211 | -0.6763 to 0.3754 | 0.467 | ns |
| FGF-21 | 0.016 | -0.5319 to 0.5540 | 0.960 | ns | -0.105 | -0.6132 to 0.4643 | 0.719 | ns | -0.085 | -0.6004 to 0.4801 | 0.772 | ns |
| FGF-23 | 0.282 | -0.3080 to 0.7156 | 0.326 | ns | -0.049 | -0.5769 to 0.5073 | 0.868 | ns | -0.318 | -0.7343 to 0.2719 | 0.266 | ns |
| FGF-5 | 0.336 | -0.2532 to 0.7434 | 0.239 | ns | -0.043 | -0.5723 to 0.5123 | 0.886 | ns | -0.381 | -0.7655 to 0.2045 | 0.179 | ns |
| Flt3L | -0.450 | -0.7981 to 0.1228 | 0.108 | ns | -0.258 | -0.7024 to 0.3318 | 0.371 | ns | 0.128 | -0.4463 to 0.6272 | 0.662 | ns |
| GDNF | 0.242 | -0.3466 to 0.6938 | 0.401 | ns | 0.016 | -0.5319 to 0.5540 | 0.960 | ns | -0.177 | -0.6569 to 0.4050 | 0.542 | ns |
| HGF | -0.235 | -0.6901 to 0.3529 | 0.415 | ns | -0.457 | -0.8012 to 0.1144 | 0.102 | ns | -0.267 | -0.7072 to 0.3232 | 0.354 | ns |
| IFN-γ | -0.007 | -0.5477 to 0.5383 | 0.985 | ns | -0.285 | -0.7168 to 0.3058 | 0.321 | ns | 0.000 | -0.5430 to 0.5430 | >0.9999 | ns |
| IL10 | 0.242 | -0.3466 to 0.6938 | 0.401 | ns | -0.206 | -0.6737 to 0.3794 | 0.476 | ns | -0.343 | -0.7468 to 0.2461 | 0.229 | ns |
| IL-10RA | 0.569 | 0.03756 to 0.8495 | 0.036 | * | -0.271 | -0.7096 to 0.3189 | 0.346 | ns | -0.520 | -0.8288 to 0.03249 | 0.059 | ns |
| IL-10RB | 0.226 | -0.3611 to 0.6851 | 0.433 | ns | -0.419 | -0.7837 to 0.1607 | 0.137 | ns | -0.540 | -0.8374 to 0.00447[illegible] | 0.049 | * |
| IL-12B | 0.208 | -0.3774 to 0.6750 | 0.471 | ns | -0.161 | -0.6476 to 0.4184 | 0.579 | ns | -0.385 | -0.7677 to 0.1995 | 0.173 | ns |
| IL13 | 0.450 | -0.1228 to 0.7981 | 0.108 | ns | -0.161 | -0.6476 to 0.4184 | 0.579 | ns | -0.264 | -0.7060 to 0.3254 | 0.358 | ns |
| IL-15RA | 0.025 | -0.5254 to 0.5602 | 0.935 | ns | -0.403 | -0.7763 to 0.1790 | 0.153 | ns | 0.011 | -0.5351 to 0.5509 | 0.972 | ns |
| IL-17A | 0.542 | -0.001309 to 0.838[illegible] | 0.048 | * | -0.058 | -0.5828 to 0.5006 | 0.844 | ns | -0.352 | -0.7512 to 0.2365 | 0.216 | ns |
| IL-17C | 0.217 | -0.3693 to 0.6801 | 0.452 | ns | -0.119 | -0.6217 to 0.4535 | 0.684 | ns | 0.011 | -0.5351 to 0.5509 | 0.972 | ns |
| IL18 | -0.197 | -0.6686 to 0.3874 | 0.496 | ns | -0.132 | -0.6300 to 0.4426 | 0.650 | ns | -0.108 | -0.6147 to 0.4625 | 0.713 | ns |
| IL-18R1 | 0.108 | -0.4625 to 0.6147 | 0.713 | ns | -0.143 | -0.6368 to 0.4334 | 0.623 | ns | -0.022 | -0.5586 to 0.5270 | 0.942 | ns |
| IL-1α | 0.293 | -0.2969 to 0.7215 | 0.306 | ns | -0.161 | -0.6476 to 0.4184 | 0.579 | ns | -0.383 | -0.7666 to 0.2020 | 0.176 | ns |
| IL2 | 0.273 | -0.3167 to 0.7108 | 0.342 | ns | -0.244 | -0.6950 to 0.3445 | 0.397 | ns | -0.302 | -0.7262 to 0.2879 | 0.291 | ns |
| IL-20 | 0.112 | -0.4589 to 0.6175 | 0.702 | ns | -0.040 | -0.5708 to 0.5139 | 0.893 | ns | -0.092 | -0.6047 to 0.4749 | 0.754 | ns |
| IL-20RA | 0.213 | -0.3734 to 0.6775 | 0.462 | ns | 0.040 | -0.5139 to 0.5708 | 0.893 | ns | -0.094 | -0.6061 to 0.4731 | 0.748 | ns |
| IL-22 RA1 | 0.345 | -0.2437 to 0.7479 | 0.226 | ns | 0.291 | -0.2991 to 0.7203 | 0.310 | ns | -0.036 | -0.5678 to 0.5172 | 0.905 | ns |
| IL-24 | 0.343 | -0.2461 to 0.7468 | 0.229 | ns | -0.199 | -0.6699 to 0.3854 | 0.491 | ns | -0.255 | -0.7012 to 0.3340 | 0.375 | ns |
| IL-2RB | 0.426 | -0.1527 to 0.7868 | 0.130 | ns | -0.166 | -0.6502 to 0.4146 | 0.568 | ns | -0.500 | -0.8201 to 0.05967 | 0.071 | ns |
| IL33 | -0.007 | -0.5477 to 0.5383 | 0.985 | ns | 0.072 | -0.4904 to 0.5917 | 0.808 | ns | 0.063 | -0.4972 to 0.5858 | 0.832 | ns |
| IL4 | -0.385 | -0.7677 to 0.1995 | 0.173 | ns | -0.383 | -0.7666 to 0.2020 | 0.176 | ns | -0.356 | -0.7535 to 0.2316 | 0.210 | ns |
| IL5 | 0.242 | -0.3466 to 0.6938 | 0.401 | ns | -0.083 | -0.5989 to 0.4818 | 0.778 | ns | -0.186 | -0.6621 to 0.3972 | 0.521 | ns |
| IL6 | 0.215 | -0.3713 to 0.6788 | 0.457 | ns | -0.025 | -0.5602 to 0.5254 | 0.935 | ns | -0.267 | -0.7072 to 0.3232 | 0.354 | ns |
| IL7 | 0.647 | 0.1609 to 0.8808 | 0.015 | * | 0.000 | -0.5430 to 0.5430 | >0.9999 | ns | -0.432 | -0.7899 to 0.1446 | 0.124 | ns |
| IL8 | 0.034 | -0.5189 to 0.5663 | 0.911 | ns | -0.213 | -0.6775 to 0.3734 | 0.462 | ns | -0.027 | -0.5617 to 0.5238 | 0.929 | ns |

| | | | | | | | | | | | | | | | | | | | | | | | | |
|---|---|---|---|---|---|---|---|---|---|---|---|---|
| LIF | 0.450 | -0.1228 to 0.7981 | 0.108 | ns | -0.123 | -0.6244 to 0.4499 | 0.673 | ns | -0.426 | -0.7868 to 0.1527 | 0.130 | ns |
| LIF-R | 0.217 | -0.3693 to 0.6801 | 0.452 | ns | -0.365 | -0.7579 to 0.2219 | 0.198 | ns | -0.354 | -0.7524 to 0.2341 | 0.213 | ns |
| MCP-1 | -0.074 | -0.5931 to 0.4887 | 0.802 | ns | -0.213 | -0.6775 to 0.3734 | 0.462 | ns | 0.094 | -0.4731 to 0.6061 | 0.748 | ns |
| MCP-2 | -0.184 | -0.6608 to 0.3991 | 0.527 | ns | -0.318 | -0.7343 to 0.2719 | 0.266 | ns | -0.110 | -0.6161 to 0.4607 | 0.707 | ns |
| MCP-3 | -0.087 | -0.6018 to 0.4783 | 0.766 | ns | -0.213 | -0.6775 to 0.3734 | 0.462 | ns | -0.316 | -0.7331 to 0.2742 | 0.269 | ns |
| MCP-4 | -0.125 | -0.6258 to 0.4481 | 0.667 | ns | -0.392 | -0.7709 to 0.1918 | 0.166 | ns | -0.040 | -0.5708 to 0.5139 | 0.893 | ns |
| MMP-1 | -0.060 | -0.5843 to 0.4989 | 0.838 | ns | -0.139 | -0.6341 to 0.4371 | 0.634 | ns | -0.132 | -0.6300 to 0.4426 | 0.650 | ns |
| MMP-10 | 0.143 | -0.4334 to 0.6368 | 0.623 | ns | -0.143 | -0.6368 to 0.4334 | 0.623 | ns | -0.300 | -0.7250 to 0.2901 | 0.295 | ns |
| NRTN | 0.224 | -0.3632 to 0.6838 | 0.438 | ns | -0.320 | -0.7354 to 0.2696 | 0.262 | ns | -0.345 | -0.7479 to 0.2437 | 0.226 | ns |
| NT-3 | 0.450 | -0.1228 to 0.7981 | 0.108 | ns | -0.300 | -0.7250 to 0.2901 | 0.295 | ns | -0.531 | -0.8336 to 0.01703 | 0.053 | ns |
| OPG | 0.412 | -0.1686 to 0.7805 | 0.144 | ns | -0.264 | -0.7060 to 0.3254 | 0.358 | ns | -0.383 | -0.7666 to 0.2020 | 0.176 | ns |
| OSM | -0.029 | -0.5632 to 0.5222 | 0.923 | ns | -0.125 | -0.6258 to 0.4481 | 0.667 | ns | -0.202 | -0.6711 to 0.3834 | 0.486 | ns |
| PD-L1 | -0.217 | -0.6801 to 0.3693 | 0.452 | ns | -0.262 | -0.7048 to 0.3275 | 0.362 | ns | 0.004 | -0.5398 to 0.5462 | 0.991 | ns |
| SCF | 0.164 | -0.4165 to 0.6489 | 0.574 | ns | -0.260 | -0.7036 to 0.3297 | 0.367 | ns | -0.307 | -0.7285 to 0.2833 | 0.284 | ns |
| SIRT2 | 0.166 | -0.4146 to 0.6502 | 0.568 | ns | -0.231 | -0.6876 to 0.3570 | 0.424 | ns | -0.264 | -0.7060 to 0.3254 | 0.358 | ns |
| SLAMF1 | 0.206 | -0.3794 to 0.6737 | 0.476 | ns | -0.385 | -0.7677 to 0.1995 | 0.173 | ns | -0.316 | -0.7331 to 0.2742 | 0.269 | ns |
| ST1A1 | 0.034 | -0.5189 to 0.5663 | 0.911 | ns | -0.117 | -0.6203 to 0.4553 | 0.690 | ns | -0.067 | -0.5887 to 0.4938 | 0.820 | ns |
| STAMBP | -0.143 | -0.6368 to 0.4334 | 0.623 | ns | -0.211 | -0.6763 to 0.3754 | 0.467 | ns | 0.134 | -0.4408 to 0.6313 | 0.645 | ns |
| TGF-α | 0.047 | -0.5090 to 0.5754 | 0.874 | ns | -0.208 | -0.6750 to 0.3774 | 0.471 | ns | -0.152 | -0.6422 to 0.4259 | 0.601 | ns |
| TGF-β1 | -0.193 | -0.6660 to 0.3913 | 0.506 | ns | 0.146 | -0.4315 to 0.6382 | 0.617 | ns | 0.193 | -0.3913 to 0.6660 | 0.506 | ns |
| TNFRSF9 | 0.112 | -0.4589 to 0.6175 | 0.702 | ns | 0.043 | -0.5123 to 0.5723 | 0.886 | ns | -0.099 | -0.6090 to 0.4696 | 0.736 | ns |
| TNFSF14 | 0.078 | -0.4853 to 0.5960 | 0.790 | ns | -0.027 | -0.5617 to 0.5238 | 0.929 | ns | -0.090 | -0.6033 to 0.4766 | 0.760 | ns |
| TNF-α | 0.237 | -0.3508 to 0.6913 | 0.410 | ns | -0.083 | -0.5989 to 0.4818 | 0.778 | ns | -0.096 | -0.6076 to 0.4713 | 0.742 | ns |
| TNF-β | 0.038 | -0.5156 to 0.5693 | 0.899 | ns | -0.515 | -0.8269 to 0.03860 | 0.062 | ns | -0.361 | -0.7557 to 0.2268 | 0.204 | ns |
| TRAIL | 0.049 | -0.5073 to 0.5769 | 0.868 | ns | 0.047 | -0.5090 to 0.5754 | 0.874 | ns | 0.065 | -0.4955 to 0.5873 | 0.826 | ns |
| TRANCE | -0.258 | -0.7024 to 0.3318 | 0.371 | ns | 0.114 | -0.4571 to 0.6189 | 0.696 | ns | -0.016 | -0.5540 to 0.5319 | 0.960 | ns |
| TSLP | 0.094 | -0.4731 to 0.6061 | 0.748 | ns | -0.085 | -0.6004 to 0.4801 | 0.772 | ns | -0.161 | -0.6476 to 0.4184 | 0.579 | ns |
| TWEAK | -0.267 | -0.7072 to 0.3232 | 0.354 | ns | 0.020 | -0.5286 to 0.5571 | 0.948 | ns | 0.202 | -0.3834 to 0.6711 | 0.486 | ns |
| uPA | -0.058 | -0.5828 to 0.5006 | 0.844 | ns | -0.036 | -0.5678 to 0.5172 | 0.905 | ns | 0.025 | -0.5254 to 0.5602 | 0.935 | ns |
| VEGFA | -0.159 | -0.6462 to 0.4203 | 0.584 | ns | 0.094 | -0.4731 to 0.6061 | 0.748 | ns | 0.253 | -0.3361 to 0.6999 | 0.379 | ns |
| β-NGF | 0.358 | -0.2292 to 0.7546 | 0.207 | ns | -0.112 | -0.6175 to 0.4589 | 0.702 | ns | -0.177 | -0.6569 to 0.4050 | 0.542 | ns |

# XY Pairs for CD8+ T cells = 14
The correlation was assessed by Spearman correaltion analysis.

**Supplementary Table 5: Correlation of protein levels in the plasma of MG patients with all 92 proteins secreted from CD4+ T cells.**

| Plasma vs | Resting CD4+ T cells | | | | Activated CD4+ T cells | | | | Ratio of activated to resting CD4+ T cells | | | |
|---|---|---|---|---|---|---|---|---|---|---|---|---|
| | Spearman r | 95% CI | P value | Signi. | Spearman r | 95% CI | P value | Signi. | Spearman r | 95% CI | P value | Signi. |
| 4E-BP1 | 0.233 | -0.3452 to 0.7883 | 0.552 | ns | 0.117 | -0.4869 to 0.5602 | 0.776 | ns | -0.083 | -0.6128 to 0.6128 | 0.843 | ns |
| ADA | 0.100 | -0.6352 to 0.5894 | 0.810 | ns | -0.133 | -0.6195 to 0.4143 | 0.744 | ns | -0.300 | -0.7682 to 0.3893 | 0.437 | ns |
| ARTN | 0.583 | -0.02600 to 0.8855 | 0.108 | ns | -0.267 | -0.7043 to 0.2814 | 0.493 | ns | -0.700 | -0.9006 to -0.04844 | 0.043 | * |
| AXIN1 | -0.467 | -0.7557 to 0.4143 | 0.213 | ns | -0.467 | -0.8042 to 0.05459 | 0.213 | ns | -0.150 | -0.7723 to 0.3807 | 0.708 | ns |
| CASP-8 | 0.133 | -0.4384 to 0.7428 | 0.744 | ns | -0.250 | -0.7681 to 0.1483 | 0.521 | ns | -0.317 | -0.8075 to 0.2978 | 0.410 | ns |
| CCL11 | -0.050 | -0.7250 to 0.4690 | 0.912 | ns | 0.067 | -0.5749 to 0.4701 | 0.880 | ns | 0.183 | -0.4462 to 0.7384 | 0.644 | ns |
| CCL19 | 0.217 | -0.3720 to 0.7763 | 0.581 | ns | -0.033 | -0.4353 to 0.6035 | 0.948 | ns | -0.117 | -0.5457 to 0.6722 | 0.776 | ns |
| CCL20 | 0.483 | -0.2362 to 0.8294 | 0.194 | ns | -0.200 | -0.5651 to 0.4813 | 0.613 | ns | -0.350 | -0.8075 to 0.2978 | 0.359 | ns |
| CCL23 | 0.100 | -0.6242 to 0.6012 | 0.810 | ns | 0.233 | -0.2957 to 0.6964 | 0.552 | ns | 0.550 | -0.1071 to 0.8666 | 0.133 | ns |
| CCL25 | -0.500 | -0.8793 to 0.05396 | 0.178 | ns | 0.267 | -0.4082 to 0.6241 | 0.493 | ns | 0.683 | 0.2014 to 0.9262 | 0.050 | ns |
| CCL28 | 0.100 | -0.5585 to 0.6619 | 0.810 | ns | 0.433 | -0.05459 to 0.8042 | 0.250 | ns | 0.083 | -0.4384 to 0.7428 | 0.843 | ns |
| CCL3 | 0.450 | -0.05396 to 0.8793 | 0.230 | ns | -0.250 | -0.6549 to 0.3638 | 0.521 | ns | -0.433 | -0.8762 to 0.06758 | 0.250 | ns |
| CCL4 | 0.200 | -0.3543 to 0.7843 | 0.613 | ns | 0.383 | -0.1609 to 0.7627 | 0.313 | ns | -0.100 | -0.7384 to 0.4462 | 0.810 | ns |
| CD244 | 0.350 | -0.2779 to 0.8149 | 0.359 | ns | 0.500 | 0.1841 to 0.8744 | 0.178 | ns | -0.183 | -0.7599 to 0.4061 | 0.644 | ns |
| CD40 | 0.017 | -0.4615 to 0.7295 | 0.982 | ns | 0.700 | 0.2650 to 0.8930 | 0.043 | * | 0.367 | -0.5325 to 0.6822 | 0.336 | ns |
| CD5 | -0.050 | -0.4764 to 0.7204 | 0.912 | ns | 0.317 | -0.2668 to 0.7122 | 0.410 | ns | 0.133 | -0.6921 to 0.5190 | 0.744 | ns |
| CD6 | 0.017 | -0.4690 to 0.7250 | 0.982 | ns | 0.317 | -0.2056 to 0.7427 | 0.410 | ns | 0.233 | -0.6185 to 0.6071 | 0.552 | ns |
| CD8A | 0.333 | -0.2469 to 0.8258 | 0.385 | ns | 0.333 | -0.1398 to 0.7716 | 0.385 | ns | -0.333 | -0.8075 to 0.2978 | 0.385 | ns |
| CDCP1 | -0.267 | -0.6722 to 0.5457 | 0.493 | ns | -0.050 | -0.5323 to 0.5166 | 0.912 | ns | -0.067 | -0.7723 to 0.3807 | 0.880 | ns |
| CSF-1 | 0.250 | -0.4061 to 0.7599 | 0.521 | ns | 0.200 | -0.4173 to 0.6173 | 0.613 | ns | 0.017 | -0.5773 to 0.6461 | 0.982 | ns |
| CST5 | -0.150 | -0.7557 to 0.4143 | 0.708 | ns | -0.300 | -0.8174 to 0.01622 | 0.437 | ns | 0.083 | -0.4764 to 0.7204 | 0.843 | ns |
| CX3CL1 | 0.533 | -0.09412 to 0.8699 | 0.148 | ns | -0.317 | -0.6984 to 0.2922 | 0.410 | ns | -0.683 | -0.9093 to -0.09650 | 0.050 | ns |
| CXCL1 | -0.633 | -0.8634 to 0.1198 | 0.076 | ns | -0.550 | -0.8254 to -0.00858 | 0.133 | ns | 0.200 | -0.3807 to 0.7723 | 0.613 | ns |
| CXCL10 | 0.667 | 0.1474 to 0.9179 | 0.059 | ns | -0.167 | -0.6883 to 0.3098 | 0.678 | ns | -0.517 | -0.8946 to -0.01785 | 0.162 | ns |
| CXCL11 | 0.200 | -0.3977 to 0.7641 | 0.613 | ns | 0.000 | -0.5676 to 0.4785 | 1.000 | ns | 0.200 | -0.4764 to 0.7204 | 0.613 | ns |
| CXCL5 | -0.150 | -0.6185 to 0.6071 | 0.708 | ns | -0.050 | -0.6418 to 0.3831 | 0.912 | ns | -0.050 | -0.6772 to 0.5391 | 0.912 | ns |
| CXCL6 | -0.333 | -0.8294 to 0.2362 | 0.385 | ns | -0.683 | -0.9244 to -0.4245 | 0.050 | ns | -0.217 | -0.7204 to 0.4764 | 0.581 | ns |
| CXCL9 | 0.650 | 0.01785 to 0.8946 | 0.067 | ns | 0.300 | -0.3028 to 0.6923 | 0.437 | ns | -0.317 | -0.6822 to 0.5325 | 0.410 | ns |
| DNER | -0.333 | -0.7682 to 0.3893 | 0.385 | ns | 0.467 | 0.1373 to 0.8625 | 0.213 | ns | 0.650 | 0.06416 to 0.9035 | 0.067 | ns |
| EN-RAGE | -0.350 | -0.8222 to 0.2574 | 0.359 | ns | -0.233 | -0.6944 to 0.2993 | 0.552 | ns | -0.617 | -0.9122 to -0.1131 | 0.086 | ns |
| FGF-19 | -0.067 | -0.6822 to 0.5325 | 0.880 | ns | 0.700 | 0.2332 to 0.8859 | 0.043 | * | 0.500 | -0.1323 to 0.8601 | 0.178 | ns |
| FGF-21 | -0.217 | -0.8222 to 0.2574 | 0.581 | ns | -0.317 | -0.6634 to 0.3507 | 0.410 | ns | -0.100 | -0.5457 to 0.6722 | 0.810 | ns |
| FGF-23 | 0.367 | -0.5121 to 0.6969 | 0.336 | ns | 0.067 | -0.5219 to 0.5271 | 0.880 | ns | -0.167 | -0.7295 to 0.4615 | 0.678 | ns |
| FGF-5 | -0.183 | -0.7843 to 0.3543 | 0.644 | ns | 0.133 | -0.4442 to 0.5964 | 0.744 | ns | 0.067 | -0.7065 to 0.4981 | 0.880 | ns |
| Flt3L | 0.350 | -0.2362 to 0.8294 | 0.359 | ns | 0.033 | -0.5245 to 0.5245 | 0.948 | ns | -0.183 | -0.7883 to 0.3452 | 0.644 | ns |
| GDNF | -0.100 | -0.6619 to 0.5585 | 0.810 | ns | 0.167 | -0.5139 to 0.5349 | 0.678 | ns | 0.283 | -0.5121 to 0.6969 | 0.463 | ns |
| HGF | -0.500 | -0.8825 to 0.04010 | 0.178 | ns | 0.000 | -0.5988 to 0.4413 | 1.000 | ns | 0.300 | -0.5711 to 0.6514 | 0.437 | ns |
| IFN-γ | 0.467 | -0.1686 to 0.8501 | 0.213 | ns | 0.400 | -0.1049 to 0.7856 | 0.291 | ns | -0.083 | -0.7384 to 0.4462 | 0.843 | ns |
| IL10 | 0.550 | -0.09412 to 0.8699 | 0.133 | ns | 0.183 | -0.5113 to 0.5375 | 0.644 | ns | 0.133 | -0.4539 to 0.7340 | 0.744 | ns |
| IL-10RA | -0.350 | -0.7922 to 0.3360 | 0.359 | ns | -0.483 | -0.8042 to 0.05459 | 0.194 | ns | -0.267 | -0.7999 to 0.3172 | 0.493 | ns |
| IL-10RB | 0.350 | -0.2469 to 0.8258 | 0.359 | ns | 0.033 | -0.3989 to 0.6308 | 0.948 | ns | -0.033 | -0.7112 to 0.4910 | 0.948 | ns |
| IL-12B | 0.483 | -0.06758 to 0.8762 | 0.194 | ns | 0.383 | -0.1225 to 0.7786 | 0.313 | ns | -0.133 | -0.6297 to 0.5953 | 0.744 | ns |
| IL13 | 0.450 | -0.1686 to 0.8501 | 0.230 | ns | 0.350 | -0.3028 to 0.6923 | 0.359 | ns | 0.333 | -0.4837 to 0.7158 | 0.385 | ns |
| IL-15RA | -0.200 | -0.7961 to 0.3267 | 0.613 | ns | 0.183 | -0.4616 to 0.5822 | 0.644 | ns | 0.283 | -0.2779 to 0.8149 | 0.463 | ns |
| IL-17A | 0.000 | -0.6822 to 0.5325 | 1.000 | ns | 0.183 | -0.3703 to 0.6505 | 0.644 | ns | 0.333 | -0.2574 to 0.8222 | 0.385 | ns |
| IL-17C | -0.367 | -0.8186 to 0.2677 | 0.336 | ns | -0.167 | -0.5773 to 0.4673 | 0.678 | ns | 0.133 | -0.4143 to 0.7557 | 0.744 | ns |
| IL18 | 0.050 | -0.4615 to 0.7295 | 0.912 | ns | -0.417 | -0.7464 to 0.1977 | 0.270 | ns | -0.617 | -0.8976 to -0.03300 | 0.086 | ns |
| IL-18R1 | -0.183 | -0.7065 to 0.4981 | 0.644 | ns | -0.033 | -0.5725 to 0.4729 | 0.948 | ns | 0.333 | -0.5258 to 0.6872 | 0.385 | ns |
| IL-1α | 0.483 | -0.1686 to 0.8501 | 0.194 | ns | 0.183 | -0.4869 to 0.5602 | 0.644 | ns | 0.183 | -0.5773 to 0.6461 | 0.644 | ns |
| IL2 | 0.883 | 0.5038 to 0.9627 | 0.003 | ** | -0.333 | -0.7083 to 0.2741 | 0.385 | ns | -0.483 | -0.7557 to 0.4143 | 0.194 | ns |
| IL-20 | -0.050 | -0.6872 to 0.5258 | 0.912 | ns | 0.383 | -0.1692 to 0.7591 | 0.313 | ns | 0.250 | -0.5258 to 0.6872 | 0.521 | ns |
| IL-20RA | 0.133 | -0.5894 to 0.6352 | 0.744 | ns | -0.483 | -0.8025 to 0.05928 | 0.194 | ns | -0.467 | -0.7682 to 0.3893 | 0.213 | ns |
| IL-22 RA1 | -0.717 | -0.9035 to -0.06416 | 0.037 | * | 0.450 | -0.3671 to 0.6527 | 0.230 | ns | 0.617 | -0.2574 to 0.8222 | 0.086 | ns |
| IL-24 | 0.100 | -0.6352 to 0.5894 | 0.810 | ns | 0.067 | -0.3895 to 0.6374 | 0.880 | ns | 0.000 | -0.6619 to 0.5585 | 1.000 | ns |
| IL-2RB | -0.250 | -0.8075 to 0.2978 | 0.521 | ns | 0.433 | -0.2922 to 0.6984 | 0.250 | ns | 0.633 | 0.1474 to 0.9179 | 0.076 | ns |
| IL33 | -0.200 | -0.6772 to 0.5391 | 0.613 | ns | 0.533 | 0.008580 to 0.8254 | 0.148 | ns | 0.500 | -0.2144 to 0.8364 | 0.178 | ns |
| IL4 | -0.083 | -0.5894 to 0.6352 | 0.843 | ns | 0.183 | -0.3339 to 0.6739 | 0.644 | ns | 0.333 | -0.04010 to 0.8825 | 0.385 | ns |
| IL5 | -0.350 | -0.8294 to 0.2362 | 0.359 | ns | 0.200 | -0.3373 to 0.6718 | 0.613 | ns | 0.200 | -0.3452 to 0.7883 | 0.613 | ns |
| IL6 | 0.683 | 0.2392 to 0.9317 | 0.050 | ns | -0.183 | -0.5627 to 0.4841 | 0.644 | ns | -0.333 | -0.7112 to 0.4910 | 0.385 | ns |
| IL7 | 0.317 | -0.2879 to 0.8112 | 0.410 | ns | 0.033 | -0.6011 to 0.4383 | 0.948 | ns | -0.167 | -0.6921 to 0.5190 | 0.678 | ns |
| IL8 | -0.050 | -0.4764 to 0.7204 | 0.912 | ns | -0.083 | -0.6330 to 0.3958 | 0.843 | ns | 0.100 | -0.7017 to 0.5051 | 0.810 | ns |

| | | | | | | | | | | | | |
|---|---|---|---|---|---|---|---|---|---|---|---|---|
| LIF | -0.133 | -0.5894 to 0.6352 | 0.744 | ns | -0.500 | -0.8381 to -0.04964 | 0.178 | ns | -0.233 | -0.6671 to 0.5521 | 0.552 | ns |
| LIF-R | -0.233 | -0.7763 to 0.3720 | 0.552 | ns | 0.550 | 0.06018 to 0.8412 | 0.133 | ns | 0.417 | 0.01785 to 0.8946 | 0.270 | ns |
| MCP-1 | 0.233 | -0.2779 to 0.8149 | 0.552 | ns | 0.150 | -0.4264 to 0.6104 | 0.708 | ns | 0.067 | -0.6671 to 0.5521 | 0.880 | ns |
| MCP-2 | -0.150 | -0.6722 to 0.5457 | 0.708 | ns | 0.083 | -0.4471 to 0.5941 | 0.843 | ns | 0.133 | -0.6128 to 0.6128 | 0.744 | ns |
| MCP-3 | -0.067 | -0.5834 to 0.6407 | 0.880 | ns | 0.433 | 0.04441 to 0.8365 | 0.250 | ns | 0.350 | -0.4462 to 0.7384 | 0.359 | ns |
| MCP-4 | 0.267 | -0.2779 to 0.8149 | 0.493 | ns | 0.133 | -0.4529 to 0.5893 | 0.744 | ns | -0.133 | -0.7557 to 0.4143 | 0.744 | ns |
| MMP-1 | -0.500 | -0.7999 to 0.3172 | 0.178 | ns | -0.183 | -0.5651 to 0.4813 | 0.644 | ns | 0.100 | -0.3720 to 0.7763 | 0.810 | ns |
| MMP-10 | 0.183 | -0.2574 to 0.8222 | 0.644 | ns | 0.317 | -0.1137 to 0.7821 | 0.410 | ns | 0.317 | -0.2254 to 0.8329 | 0.410 | ns |
| NRTN | -0.167 | -0.7763 to 0.3720 | 0.678 | ns | -0.133 | -0.6944 to 0.2993 | 0.744 | ns | 0.017 | -0.5521 to 0.6671 | 0.982 | ns |
| NT-3 | 0.200 | -0.4615 to 0.7295 | 0.613 | ns | -0.383 | -0.7445 to 0.2017 | 0.313 | ns | -0.317 | -0.7843 to 0.3543 | 0.410 | ns |
| OPG | 0.850 | 0.4546 to 0.9577 | 0.006 | ** | 0.467 | -0.04514 to 0.8075 | 0.213 | ns | -0.317 | -0.7295 to 0.4615 | 0.410 | ns |
| OSM | 0.183 | -0.4690 to 0.7250 | 0.644 | ns | -0.400 | -0.8025 to 0.05928 | 0.291 | ns | -0.350 | -0.8112 to 0.2879 | 0.359 | ns |
| PD-L1 | -0.233 | -0.6772 to 0.5391 | 0.552 | ns | 0.183 | -0.4051 to 0.6263 | 0.644 | ns | 0.217 | -0.5585 to 0.6619 | 0.581 | ns |
| SCF | 0.550 | -0.1803 to 0.8468 | 0.133 | ns | 0.483 | -0.04988 to 0.8059 | 0.194 | ns | 0.150 | -0.4690 to 0.7250 | 0.708 | ns |
| SIRT2 | 0.200 | -0.4143 to 0.7557 | 0.613 | ns | 0.000 | -0.6374 to 0.3895 | 1.000 | ns | -0.117 | -0.7471 to 0.4305 | 0.776 | ns |
| SLAMF1 | 0.083 | -0.6012 to 0.6242 | 0.843 | ns | 0.183 | -0.3989 to 0.6308 | 0.644 | ns | 0.017 | -0.6514 to 0.5711 | 0.982 | ns |
| ST1A1 | -0.433 | -0.8699 to 0.09412 | 0.250 | ns | -0.600 | -0.8916 to -0.2585 | 0.097 | ns | -0.400 | -0.8364 to 0.2144 | 0.291 | ns |
| STAMBP | -0.133 | -0.6407 to 0.5834 | 0.744 | ns | 0.150 | -0.5451 to 0.5032 | 0.708 | ns | 0.083 | -0.5325 to 0.6822 | 0.843 | ns |
| TGF-α | 0.133 | -0.5773 to 0.6461 | 0.744 | ns | 0.317 | -0.1567 to 0.7645 | 0.410 | ns | 0.333 | -0.4061 to 0.7599 | 0.385 | ns |
| TGF-β1 | -0.183 | -0.6407 to 0.5834 | 0.644 | ns | 0.217 | -0.3735 to 0.6484 | 0.581 | ns | 0.317 | -0.4981 to 0.7065 | 0.410 | ns |
| TNFRSF9 | 0.783 | 0.3411 to 0.9449 | 0.017 | * | 0.167 | -0.3028 to 0.6923 | 0.678 | ns | -0.150 | -0.5953 to 0.6297 | 0.708 | ns |
| TNFSF14 | -0.183 | -0.6514 to 0.5711 | 0.644 | ns | 0.150 | -0.4442 to 0.5964 | 0.708 | ns | 0.283 | -0.5391 to 0.6772 | 0.463 | ns |
| TNF-α | 0.367 | -0.2254 to 0.8329 | 0.336 | ns | -0.017 | -0.4729 to 0.5725 | 0.982 | ns | -0.350 | -0.7557 to 0.4143 | 0.359 | ns |
| TNF-β | 0.200 | -0.3360 to 0.7922 | 0.613 | ns | 0.250 | -0.3339 to 0.6739 | 0.521 | ns | 0.250 | -0.5894 to 0.6352 | 0.521 | ns |
| TRAIL | -0.067 | -0.5391 to 0.6772 | 0.880 | ns | 0.300 | -0.3407 to 0.6697 | 0.437 | ns | 0.300 | -0.4837 to 0.7158 | 0.437 | ns |
| TRANCE | 0.017 | -0.3632 to 0.7804 | 0.982 | ns | -0.283 | -0.6944 to 0.2993 | 0.463 | ns | 0.033 | -0.7112 to 0.4910 | 0.948 | ns |
| TSLP | -0.133 | -0.5258 to 0.6872 | 0.744 | ns | 0.267 | -0.3440 to 0.6676 | 0.493 | ns | 0.333 | -0.5894 to 0.6352 | 0.385 | ns |
| TWEAK | 0.283 | -0.5457 to 0.6722 | 0.463 | ns | 0.500 | -0.05928 to 0.8025 | 0.178 | ns | -0.017 | -0.4837 to 0.7158 | 0.982 | ns |
| uPA | 0.300 | -0.4837 to 0.7158 | 0.437 | ns | 0.000 | -0.4785 to 0.5676 | 1.000 | ns | -0.033 | -0.5834 to 0.6407 | 0.948 | ns |
| VEGFA | 0.400 | -0.5121 to 0.6969 | 0.291 | ns | -0.150 | -0.7609 to 0.1651 | 0.708 | ns | -0.783 | -0.9527 to -0.4077 | 0.017 | * |
| β-NGF | -0.083 | -0.5894 to 0.6352 | 0.843 | ns | 0.767 | 0.4320 to 0.9257 | 0.021 | * | 0.850 | 0.5293 to 0.9651 | 0.006 | ** |

# XY Pairs for plasma and CD4+ T cells = 9

The correlation was assessed by Spearman correaltion analysis.

**Supplementary Table 6: Correlation of protein levels in the serum of MG patients with all 92 proteins secreted from CD4+ T cells.**

| Serum vs | Resting CD4+ T cells | | | | Activated CD4+ T cells | | | | Ratio of activated to resting CD4+ T cells | | | |
|---|---|---|---|---|---|---|---|---|---|---|---|---|
| | Spearman r | 95% CI | P value | Signi. | Spearman r | 95% CI | P value | Signi. | Spearman r | 95% CI | P value | Signi. |
| 4E-BP1 | 0.017 | -0.6145 to 0.5081 | 0.982 | ns | -0.433 | -0.7683 to 0.2543 | 0.250 | ns | -0.400 | -0.8037 to 0.1657 | 0.291 | ns |
| ADA | 0.333 | -0.2483 to 0.7710 | 0.385 | ns | -0.033 | -0.4518 to 0.6580 | 0.948 | ns | -0.283 | -0.7710 to 0.2483 | 0.463 | ns |
| ARTN | -0.083 | -0.6482 to 0.4652 | 0.843 | ns | -0.333 | -0.7631 to 0.2661 | 0.385 | ns | -0.267 | -0.6580 to 0.4518 | 0.493 | ns |
| AXIN1 | -0.317 | -0.7189 to 0.3547 | 0.410 | ns | -0.383 | -0.8061 to 0.1590 | 0.313 | ns | -0.117 | -0.7102 to 0.3702 | 0.776 | ns |
| CASP-8 | 0.083 | -0.3853 to 0.7014 | 0.843 | ns | 0.333 | -0.3282 to 0.7331 | 0.385 | ns | 0.250 | -0.2602 to 0.7657 | 0.521 | ns |
| CCL11 | -0.167 | -0.7863 to 0.2112 | 0.678 | ns | -0.200 | -0.7710 to 0.2483 | 0.613 | ns | 0.200 | -0.4241 to 0.6770 | 0.613 | ns |
| CCL19 | -0.200 | -0.4741 to 0.6416 | 0.613 | ns | -0.467 | -0.7657 to 0.2602 | 0.213 | ns | -0.117 | -0.6984 to 0.3903 | 0.776 | ns |
| CCL20 | -0.017 | -0.5859 to 0.5404 | 0.982 | ns | -0.250 | -0.6548 to 0.4563 | 0.521 | ns | -0.200 | -0.7189 to 0.3547 | 0.613 | ns |
| CCL23 | -0.150 | -0.6801 to 0.4194 | 0.708 | ns | -0.050 | -0.6110 to 0.5122 | 0.912 | ns | 0.667 | 0.2461 to 0.9100 | 0.059 | ns |
| CCL25 | -0.600 | -0.8133 to 0.1385 | 0.097 | ns | 0.100 | -0.5786 to 0.5483 | 0.810 | ns | 0.433 | -0.2238 to 0.7813 | 0.250 | ns |
| CCL28 | 0.644 | 0.2323 to 0.9074 | 0.068 | ns | 0.209 | -0.2200 to 0.7828 | 0.589 | ns | -0.293 | -0.7959 to 0.1867 | 0.442 | ns |
| CCL3 | 0.517 | -0.1985 to 0.7914 | 0.162 | ns | -0.167 | -0.6145 to 0.5081 | 0.678 | ns | -0.450 | -0.6580 to 0.4518 | 0.230 | ns |
| CCL4 | 0.267 | -0.4741 to 0.6416 | 0.493 | ns | 0.533 | -0.07375 to 0.8344 | 0.148 | ns | -0.100 | -0.4652 to 0.6482 | 0.810 | ns |
| CD244 | 0.133 | -0.4288 to 0.6739 | 0.744 | ns | 0.150 | -0.3173 to 0.7387 | 0.708 | ns | -0.017 | -0.4913 to 0.6282 | 0.982 | ns |
| CD40 | -0.683 | -0.8717 to -0.06367 | 0.050 | ns | 0.083 | -0.4194 to 0.6801 | 0.843 | ns | 0.817 | 0.5222 to 0.9523 | 0.011 | * |
| CD5 | 0.100 | -0.3952 to 0.6954 | 0.810 | ns | 0.467 | -0.1454 to 0.8110 | 0.213 | ns | 0.117 | -0.5325 to 0.5932 | 0.776 | ns |
| CD6 | 0.333 | -0.1454 to 0.8110 | 0.385 | ns | 0.883 | 0.7304 to 0.9760 | 0.003 | ** | 0.300 | -0.2778 to 0.7578 | 0.437 | ns |
| CD8A | -0.133 | -0.7131 to 0.3651 | 0.744 | ns | -0.133 | -0.6770 to 0.4241 | 0.744 | ns | -0.117 | -0.5325 to 0.5932 | 0.776 | ns |
| CDCP1 | -0.317 | -0.6482 to 0.4652 | 0.410 | ns | -0.333 | -0.7303 to 0.3335 | 0.385 | ns | -0.117 | -0.6482 to 0.4652 | 0.776 | ns |
| CSF-1 | -0.100 | -0.4241 to 0.6770 | 0.810 | ns | 0.217 | -0.3227 to 0.7359 | 0.581 | ns | 0.417 | -0.1522 to 0.8085 | 0.270 | ns |
| CST5 | -0.133 | -0.6863 to 0.4098 | 0.744 | ns | -0.233 | -0.7736 to 0.2422 | 0.552 | ns | 0.033 | -0.6644 to 0.4427 | 0.948 | ns |
| CX3CL1 | -0.217 | -0.5823 to 0.5444 | 0.581 | ns | 0.100 | -0.5560 to 0.5712 | 0.810 | ns | 0.283 | -0.4146 to 0.6832 | 0.463 | ns |
| CXCL1 | -0.417 | -0.6770 to 0.4241 | 0.270 | ns | -0.267 | -0.6110 to 0.5122 | 0.493 | ns | -0.117 | -0.5365 to 0.5896 | 0.776 | ns |
| CXCL10 | 0.017 | -0.5164 to 0.6075 | 0.982 | ns | 0.000 | -0.6039 to 0.5204 | 1.000 | ns | 0.067 | -0.4335 to 0.6707 | 0.880 | ns |
| CXCL11 | 0.367 | -0.1454 to 0.8110 | 0.336 | ns | 0.367 | -0.3442 to 0.7247 | 0.336 | ns | -0.033 | -0.6580 to 0.4518 | 0.948 | ns |
| CXCL5 | -0.383 | -0.6954 to 0.3952 | 0.313 | ns | 0.033 | -0.5122 to 0.6110 | 0.948 | ns | 0.383 | -0.08858 to 0.8298 | 0.313 | ns |
| CXCL6 | -0.017 | -0.6449 to 0.4697 | 0.982 | ns | -0.333 | -0.7631 to 0.2661 | 0.385 | ns | -0.483 | -0.7939 to 0.1921 | 0.194 | ns |
| CXCL9 | 0.650 | 0.2356 to 0.9081 | 0.067 | ns | 0.067 | -0.5598 to 0.5674 | 0.880 | ns | -0.567 | -0.8963 to -0.1749 | 0.121 | ns |
| DNER | -0.350 | -0.6548 to 0.4563 | 0.359 | ns | 0.350 | -0.08120 to 0.8321 | 0.359 | ns | 0.567 | 0.05497 to 0.8696 | 0.121 | ns |
| EN-RAGE | 0.317 | -0.1657 to 0.8037 | 0.410 | ns | 0.533 | -0.02766 to 0.8479 | 0.148 | ns | 0.617 | 0.2253 to 0.9061 | 0.086 | ns |
| FGF-19 | -0.050 | -0.6179 to 0.5040 | 0.912 | ns | 0.417 | -0.1856 to 0.7963 | 0.270 | ns | 0.283 | -0.3853 to 0.7014 | 0.463 | ns |
| FGF-21 | -0.217 | -0.7939 to 0.1921 | 0.581 | ns | -0.267 | -0.6449 to 0.4697 | 0.493 | ns | -0.083 | -0.6770 to 0.4241 | 0.843 | ns |
| FGF-23 | 0.167 | -0.5932 to 0.5325 | 0.678 | ns | -0.133 | -0.6676 to 0.4381 | 0.744 | ns | -0.317 | -0.5674 to 0.5598 | 0.410 | ns |
| FGF-5 | 0.233 | -0.5245 to 0.6004 | 0.552 | ns | -0.483 | -0.8545 to 0.00366 | 0.194 | ns | -0.567 | -0.8181 to 0.1245 | 0.121 | ns |
| Flt3L | -0.033 | -0.4697 to 0.6449 | 0.948 | ns | -0.133 | -0.6644 to 0.4427 | 0.744 | ns | -0.017 | -0.4473 to 0.6612 | 0.982 | ns |
| GDNF | -0.067 | -0.6110 to 0.5122 | 0.880 | ns | -0.283 | -0.7387 to 0.3173 | 0.463 | ns | -0.150 | -0.6282 to 0.4913 | 0.708 | ns |
| HGF | -0.267 | -0.7683 to 0.2543 | 0.493 | ns | 0.217 | -0.3753 to 0.7073 | 0.581 | ns | 0.100 | -0.2483 to 0.7710 | 0.810 | ns |
| IFN-γ | 0.283 | -0.3651 to 0.7131 | 0.463 | ns | 0.417 | -0.1175 to 0.8205 | 0.270 | ns | 0.133 | -0.3702 to 0.7102 | 0.744 | ns |
| IL10 | 0.567 | 0.07245 to 0.8738 | 0.121 | ns | -0.117 | -0.7218 to 0.3495 | 0.776 | ns | -0.217 | -0.7247 to 0.3442 | 0.581 | ns |
| IL-10RA | -0.533 | -0.8632 to -0.02937 | 0.148 | ns | -0.633 | -0.8902 to -0.1458 | 0.076 | ns | -0.217 | -0.5896 to 0.5365 | 0.581 | ns |
| IL-10RB | 0.700 | 0.2568 to 0.9119 | 0.043 | * | -0.167 | -0.5968 to 0.5285 | 0.678 | ns | -0.233 | -0.6515 to 0.4608 | 0.552 | ns |
| IL-12B | 0.483 | -0.1522 to 0.8085 | 0.194 | ns | 0.100 | -0.4146 to 0.6832 | 0.810 | ns | -0.350 | -0.7524 to 0.2893 | 0.359 | ns |
| IL13 | 0.167 | -0.5560 to 0.5712 | 0.678 | ns | 0.250 | -0.3702 to 0.7102 | 0.521 | ns | 0.283 | -0.1454 to 0.8110 | 0.463 | ns |
| IL-15RA | 0.167 | -0.4913 to 0.6282 | 0.678 | ns | -0.200 | -0.6612 to 0.4473 | 0.613 | ns | -0.133 | -0.6863 to 0.4098 | 0.744 | ns |
| IL-17A | -0.233 | -0.7303 to 0.3335 | 0.552 | ns | 0.333 | -0.09590 to 0.8275 | 0.385 | ns | 0.417 | -0.4146 to 0.6832 | 0.270 | ns |
| IL-17C | 0.100 | -0.3952 to 0.6954 | 0.810 | ns | -0.500 | -0.8412 to 0.05101 | 0.178 | ns | -0.267 | -0.8434 to 0.04330 | 0.493 | ns |
| IL18 | -0.133 | -0.5404 to 0.5859 | 0.744 | ns | -0.650 | -0.9081 to -0.2356 | 0.067 | ns | -0.583 | -0.8366 to 0.06624 | 0.108 | ns |
| IL-18R1 | -0.317 | -0.6707 to 0.4335 | 0.410 | ns | -0.133 | -0.6580 to 0.4518 | 0.744 | ns | 0.250 | -0.3389 to 0.7275 | 0.521 | ns |
| IL-1α | -0.217 | -0.5245 to 0.6004 | 0.581 | ns | -0.567 | -0.8696 to -0.05497 | 0.121 | ns | -0.567 | -0.7247 to 0.3442 | 0.121 | ns |
| IL2 | -0.383 | -0.7761 to 0.2361 | 0.313 | ns | 0.450 | -0.1103 to 0.8228 | 0.230 | ns | 0.567 | 0.2048 to 0.9022 | 0.121 | ns |
| IL-20 | 0.400 | -0.1385 to 0.8133 | 0.291 | ns | 0.600 | 0.2253 to 0.9061 | 0.097 | ns | 0.183 | -0.4335 to 0.6707 | 0.644 | ns |
| IL-20RA | -0.150 | -0.7470 to 0.3006 | 0.708 | ns | 0.583 | -0.1657 to 0.8037 | 0.108 | ns | 0.650 | 0.3343 to 0.9252 | 0.067 | ns |
| IL-22 RA1 | 0.033 | -0.6548 to 0.4563 | 0.948 | ns | 0.083 | -0.5859 to 0.5404 | 0.843 | ns | -0.167 | -0.5674 to 0.5598 | 0.678 | ns |
| IL-24 | 0.550 | 0.1084 to 0.8821 | 0.133 | ns | 0.283 | -0.2720 to 0.7604 | 0.463 | ns | 0.117 | -0.3853 to 0.7014 | 0.776 | ns |
| IL-2RB | -0.050 | -0.4784 to 0.6383 | 0.912 | ns | 0.217 | -0.2422 to 0.7736 | 0.581 | ns | 0.433 | -0.1175 to 0.8205 | 0.250 | ns |
| IL33 | -0.417 | -0.8479 to 0.02766 | 0.270 | ns | 0.333 | -0.1245 to 0.8181 | 0.385 | ns | 0.450 | -0.1724 to 0.8013 | 0.230 | ns |
| IL4 | -0.067 | -0.5365 to 0.5896 | 0.880 | ns | -0.317 | -0.6801 to 0.4194 | 0.410 | ns | -0.133 | -0.7014 to 0.3853 | 0.744 | ns |
| IL5 | 0.183 | -0.5365 to 0.5896 | 0.644 | ns | 0.250 | -0.2175 to 0.7838 | 0.521 | ns | 0.167 | -0.3173 to 0.7387 | 0.678 | ns |
| IL6 | 0.400 | 0.02100 to 0.8610 | 0.291 | ns | -0.167 | -0.5040 to 0.6179 | 0.678 | ns | -0.117 | -0.5636 to 0.5636 | 0.776 | ns |
| IL7 | 0.483 | -0.3227 to 0.7359 | 0.194 | ns | 0.283 | -0.3702 to 0.7102 | 0.463 | ns | 0.017 | -0.4473 to 0.6612 | 0.982 | ns |
| IL8 | 0.267 | -0.2836 to 0.7551 | 0.493 | ns | -0.617 | -0.8567 to -0.0044 | 0.086 | ns | -0.267 | -0.7524 to 0.2893 | 0.493 | ns |

| | | | | | | | | | | | | |
|---|---|---|---|---|---|---|---|---|---|---|---|---|
| LIF | -0.417 | -0.8275 to 0.09590 | 0.270 | ns | -0.133 | -0.4518 to 0.6580 | 0.744 | ns | 0.117 | -0.4784 to 0.6383 | 0.776 | ns |
| LIF-R | 0.433 | -0.4518 to 0.6580 | 0.250 | ns | 0.300 | -0.3753 to 0.7073 | 0.437 | ns | -0.217 | -0.7939 to 0.1921 | 0.581 | ns |
| MCP-1 | -0.317 | -0.6179 to 0.5040 | 0.410 | ns | -0.067 | -0.7131 to 0.3651 | 0.880 | ns | 0.400 | -0.1175 to 0.8205 | 0.291 | ns |
| MCP-2 | -0.417 | -0.8501 to 0.01973 | 0.270 | ns | -0.217 | -0.6350 to 0.4827 | 0.581 | ns | 0.000 | -0.5285 to 0.5968 | 1.000 | ns |
| MCP-3 | 0.567 | 0.02100 to 0.8610 | 0.121 | ns | 0.000 | -0.6145 to 0.5081 | 1.000 | ns | -0.200 | -0.6179 to 0.5040 | 0.613 | ns |
| MCP-4 | 0.183 | -0.1103 to 0.8228 | 0.644 | ns | -0.300 | -0.7761 to 0.2361 | 0.437 | ns | -0.217 | -0.6482 to 0.4652 | 0.581 | ns |
| MMP-1 | 0.200 | -0.2300 to 0.7787 | 0.613 | ns | 0.117 | -0.4050 to 0.6893 | 0.776 | ns | 0.050 | -0.4956 to 0.6248 | 0.912 | ns |
| MMP-10 | -0.067 | -0.4913 to 0.6282 | 0.880 | ns | 0.233 | -0.1657 to 0.8037 | 0.552 | ns | 0.233 | -0.3389 to 0.7275 | 0.552 | ns |
| NRTN | 0.033 | -0.6707 to 0.4335 | 0.948 | ns | -0.017 | -0.6075 to 0.5164 | 0.982 | ns | -0.050 | -0.4098 to 0.6863 | 0.912 | ns |
| NT-3 | 0.383 | -0.2422 to 0.7736 | 0.313 | ns | -0.183 | -0.6548 to 0.4563 | 0.644 | ns | -0.367 | -0.7787 to 0.2300 | 0.336 | ns |
| OPG | 0.633 | 0.2356 to 0.9081 | 0.076 | ns | 0.267 | -0.2361 to 0.7761 | 0.493 | ns | -0.333 | -0.7524 to 0.2893 | 0.385 | ns |
| OSM | -0.400 | -0.7551 to 0.2836 | 0.291 | ns | -0.200 | -0.6676 to 0.4381 | 0.613 | ns | 0.133 | -0.4001 to 0.6923 | 0.744 | ns |
| PD-L1 | -0.417 | -0.6984 to 0.3903 | 0.270 | ns | -0.250 | -0.5968 to 0.5285 | 0.521 | ns | 0.083 | -0.3803 to 0.7043 | 0.843 | ns |
| SCF | 0.083 | -0.4050 to 0.6893 | 0.843 | ns | -0.083 | -0.6316 to 0.4870 | 0.843 | ns | -0.200 | -0.7863 to 0.2112 | 0.613 | ns |
| SIRT2 | 0.017 | -0.5712 to 0.5560 | 0.982 | ns | -0.217 | -0.7415 to 0.3117 | 0.581 | ns | -0.367 | -0.7604 to 0.2720 | 0.336 | ns |
| SLAMF1 | -0.133 | -0.6515 to 0.4608 | 0.744 | ns | 0.250 | -0.1454 to 0.8110 | 0.521 | ns | 0.133 | -0.5040 to 0.6179 | 0.744 | ns |
| ST1A1 | 0.033 | -0.5521 to 0.5749 | 0.948 | ns | 0.600 | -0.1103 to 0.8228 | 0.097 | ns | 0.650 | 0.3004 to 0.9196 | 0.067 | ns |
| STAMBP | -0.100 | -0.7218 to 0.3495 | 0.810 | ns | -0.150 | -0.6350 to 0.4827 | 0.708 | ns | -0.233 | -0.7415 to 0.3117 | 0.552 | ns |
| TGF-α | -0.333 | -0.6350 to 0.4827 | 0.385 | ns | -0.550 | -0.7838 to 0.2175 | 0.133 | ns | -0.200 | -0.6110 to 0.5122 | 0.613 | ns |
| TGF-β1 | -0.133 | -0.6612 to 0.4473 | 0.744 | ns | 0.650 | 0.02937 to 0.8632 | 0.067 | ns | 0.767 | 0.3934 to 0.9344 | 0.021 | * |
| TNFRSF9 | 0.683 | 0.1084 to 0.8821 | 0.050 | ns | 0.067 | -0.4241 to 0.6770 | 0.880 | ns | -0.150 | -0.7102 to 0.3702 | 0.708 | ns |
| TNFSF14 | -0.033 | -0.6316 to 0.4870 | 0.948 | ns | 0.483 | -0.2049 to 0.7889 | 0.194 | ns | 0.200 | -0.2602 to 0.7657 | 0.613 | ns |
| TNF-α | -0.300 | -0.6548 to 0.4563 | 0.437 | ns | 0.000 | -0.3702 to 0.7102 | 1.000 | ns | 0.050 | -0.4050 to 0.6893 | 0.912 | ns |
| TNF-β | -0.067 | -0.5245 to 0.6004 | 0.880 | ns | 0.167 | -0.3335 to 0.7303 | 0.678 | ns | 0.283 | -0.2049 to 0.7889 | 0.463 | ns |
| TRAIL | -0.217 | -0.6612 to 0.4473 | 0.581 | ns | -0.133 | -0.7497 to 0.2950 | 0.744 | ns | 0.133 | -0.4563 to 0.6548 | 0.744 | ns |
| TRANCE | 0.200 | -0.2112 to 0.7863 | 0.613 | ns | -0.250 | -0.6954 to 0.3952 | 0.521 | ns | -0.067 | -0.6416 to 0.4741 | 0.880 | ns |
| TSLP | 0.100 | -0.4697 to 0.6449 | 0.810 | ns | -0.117 | -0.7043 to 0.3803 | 0.776 | ns | 0.000 | -0.3651 to 0.7131 | 1.000 | ns |
| TWEAK | -0.417 | -0.7415 to 0.3117 | 0.270 | ns | 0.317 | -0.2543 to 0.7683 | 0.410 | ns | 0.567 | -0.06624 to 0.8366 | 0.121 | ns |
| uPA | -0.033 | -0.6739 to 0.4288 | 0.948 | ns | 0.100 | -0.4652 to 0.6482 | 0.810 | ns | 0.000 | -0.4697 to 0.6449 | 1.000 | ns |
| VEGFA | 0.800 | 0.3004 to 0.9196 | 0.014 | * | 0.533 | -0.3282 to 0.7331 | 0.148 | ns | -0.233 | -0.6248 to 0.4956 | 0.552 | ns |
| β-NGF | -0.050 | -0.4652 to 0.6482 | 0.912 | ns | 0.617 | 0.1947 to 0.9002 | 0.086 | ns | 0.600 | 0.004481 to 0.8567 | 0.097 | ns |

# XY Pairs for serum and CD4+ T cells = 9

The correlation was assessed by Spearman correaltion analysis.

**Supplementary Table 7: T cells secreted proteins significantly differed in MG patients based on their clinical visits**

| Resting CD4+ T cells (1st vs follow-up visits) | | |
|---|---|---|
| | P value | FDR q Value |
| 4E-BP1 | 0.0004 | 0.0013 |
| ADA | 0.0015 | 0.0027 |
| AXIN1 | 0.0013 | 0.0027 |
| CASP-8 | 0.0003 | 0.0013 |
| STAMBP | 0.0001 | 0.0011 |
| TGF-β1 | 0.0003 | 0.0013 |
| TNFSF14 | 0.0040 | 0.0043 |
| TRAIL | 0.0089 | 0.0089 |
| CD5 | 0.0019 | 0.0027 |
| CD6 | 0.0028 | 0.0036 |
| Flt3L | 0.0016 | 0.0027 |
| IL18 | 0.0006 | 0.0014 |
| IL-18R1 | 0.0031 | 0.0036 |

| Activated CD4+ T cells (1st vs follow-up visits) | | |
|---|---|---|
| | P value | FDR q Value |
| 4E-BP1 | 0.0041 | 0.0053 |
| ADA | 0.0015 | 0.0029 |
| AXIN1 | 0.0041 | 0.0053 |
| CCL20 | 0.0013 | 0.0029 |
| CD5 | 0.0018 | 0.0032 |
| CD6 | 0.0026 | 0.0039 |
| CX3CL1 | 0.0097 | 0.0110 |
| CXCL11 | 0.0001 | 0.0011 |
| CASP-8 | 0.0002 | 0.0012 |
| IL2 | 0.0001 | 0.0011 |
| IL4 | 0.0003 | 0.0012 |
| IL-24 | 0.0003 | 0.0012 |
| LIF | 0.0145 | 0.0153 |
| NT-3 | 0.0188 | 0.0188 |
| SIRT2 | 0.0083 | 0.0100 |
| STAMBP | 0.0009 | 0.0023 |
| TRANCE | 0.0004 | 0.0013 |
| TWEAK | 0.0026 | 0.0039 |

| Activated/Resting CD4+ T cells (1st vs follow-up visits) | | |
|---|---|---|
| | P value | FDR q Value |
| Flt3L | 0.0014 | 0.0026 |
| IL-10RA | 0.0115 | 0.0129 |
| IL13 | 0.0932 | 0.0932 |
| IL-18R1 | 0.0006 | 0.0019 |
| IL2 | 0.0048 | 0.0062 |
| IL4 | 0.0020 | 0.0030 |
| TGF-β1 | 0.0008 | 0.0019 |
| TNFSF14 | 0.0008 | 0.0019 |
| TRANCE | 0.0001 | 0.0006 |

Proteins confirmed in Boruta algorithm are varified by unpaired t test with Welch correction and false discovery rate method by Benjamini and Hochberg. n = 8 MG patients, 11 HCs for CD4+ T cells.
